# Factors affecting hesitancy toward COVID-19 vaccine booster doses in Canada: a cross-national survey

**DOI:** 10.17269/s41997-023-00823-z

**Published:** 2023-11-22

**Authors:** Jeanna Parsons Leigh, Emily A. FitzGerald, Stephana J. Moss, Rebecca Brundin-Mather, Alexandra Dodds, Henry T. Stelfox, Ève Dubé, Kirsten M. Fiest, Donna Halperin, Sofia B. Ahmed, Shannon E. MacDonald, Sharon E. Straus, Terra Manca, Josh Ng Kamstra, Andrea Soo, Shelly Longmore, Shelly Kupsch, Bonnie Sept, Scott Halperin

**Affiliations:** 1 Faculty of Health, School of Health Administration, Dalhousie University, Halifax, NS Canada; 2 Department of Critical Care Medicine, Dalhousie University, Halifax, NS Canada; 3 Canadian Center for Vaccinology & IWK Health Centre, Halifax, NS Canada; 4 CRISMA Center, Department of Critical Care, University of Pittsburgh, Pittsburgh, USA; 5 Department of Critical Care Medicine, University of Calgary, Calgary, AB Canada; 6 O’Brien Institute for Public Health, University of Calgary, Calgary, AB Canada; 7 Centre de Recherche du CHU de Québec, Université Laval, Québec, Canada; 8 Institut National de Santé Publique du Québec, Québec, Canada; 9 Department of Psychiatry & Hotchkiss Brain Institute, Cumming School of Medicine, University of Calgary, Calgary, AB Canada; 10 Rankin School of Nursing, St. Francis Xavier University, Antigonish, NS Canada; 11 Department of Medicine, University of Calgary, Calgary, AB Canada; 12 Libin Cardiovascular Institute, University of Calgary, Calgary, AB Canada; 13 School of Public Health, University of Alberta, Edmonton, AB Canada; 14 Faculty of Nursing, University of Alberta, Edmonton, AB Canada; 15 Li Ka Shing Knowledge Institute of St. Michael’s Hospital, Unity Health Toronto, Toronto, ON Canada; 16 Department of Medicine, University of Toronto, Toronto, ON Canada; 17 Institute of Health Policy, Management, and Evaluation, University of Toronto, Toronto, ON Canada; 18 Sociology and Social Anthropology, Dalhousie University, Halifax, NS Canada; 19 Department of Surgery, John A. Burns School of Medicine, University of Hawaii at Manoa, Honolulu, HI USA; 20 Department of Surgery, The Queen’s Medical Center, Honolulu, HI USA; 21 Alberta Health Services, Calgary, AB Canada; 22 Department of Pediatrics, Faculty of Medicine, Dalhousie University, Halifax, NS Canada; 23 Department of Microbiology and Immunology, Faculty of Medicine, Dalhousie University, Halifax, NS Canada

**Keywords:** Vaccine hesitancy, Vaccination, COVID-19, SARS-CoV-2, Survey, Questionnaire, Hésitation à la vaccination, vaccination, COVID-19, SRAS-CoV-2, enquête, questionnaire

## Abstract

**Objective:**

COVID-19 transmission, emergence of variants of concern, and weakened immunity have led to recommended vaccine booster doses for COVID-19. Vaccine hesitancy challenges broad immunization coverage. We deployed a cross-national survey to investigate knowledge, beliefs, and behaviours toward continued COVID-19 vaccination.

**Methods:**

We administered a national, cross-sectional online survey among adults in Canada between March 16 and March 26, 2022. We utilized descriptive statistics to summarize our sample, and tested for demographic differences, perceptions of vaccine effectiveness, recommended doses, and trust in decisions, using the Rao-Scott correction for weighted chi-squared tests. Multivariable logistic regression was adjusted for relevant covariates to identify sociodemographic factors and beliefs associated with vaccine hesitancy.

**Results:**

We collected 2202 completed questionnaires. Lower education status (high school: odds ratio (OR) 1.90, 95% confidence interval (CI) 1.29, 2.81) and having children (OR 1.89, CI 1.39, 2.57) were associated with increased odds of experiencing hesitancy toward a booster dose, while higher income ($100,000–$149,999: OR 0.60, CI 0.39, 0.91; $150,000 or more: OR 0.49, CI 0.29, 0.82) was associated with decreased odds. Disbelief in vaccine effectiveness (against infection: OR 3.69, CI 1.98, 6.90; serious illness: OR 3.15, CI 1.69, 5.86), disagreeing with government decision-making (somewhat disagree: OR 2.70, CI 1.38, 5.29; strongly disagree: OR 4.62, CI 2.20, 9.7), and beliefs in over-vaccinating (OR 2.07, CI 1.53, 2.80) were found associated with booster dose hesitancy.

**Conclusion:**

COVID-19 vaccine hesitancy may develop or increase regarding subsequent vaccines. Our findings indicate factors to consider when targeting vaccine-hesitant populations.

**Supplementary Information:**

The online version contains supplementary material available at 10.17269/s41997-023-00823-z.

## Résumé

**Objectif:**

La transmission de la COVID-19, l’émergence de variants préoccupants et l’affaiblissement de l’immunité ont conduit à recommander des doses de rappel de vaccin contre la COVID-19. L’hésitation à la vaccination remet en question une large couverture vaccinale. Nous avons déployé une enquête transnationale pour étudier les connaissances, les croyances et les comportements en faveur de la poursuite de la vaccination contre la COVID-19.

**Méthodes:**

Nous avons mené une enquête nationale transversale en ligne auprès d’adultes au Canada, entre le 16 et le 26 mars 2022. Nous avons utilisé des statistiques descriptives pour résumer notre échantillon et testé les différences démographiques, les perceptions de l’efficacité des vaccins, les doses recommandées et la confiance dans les décisions, en utilisant la correction de Rao-Scott pour les tests du chi carré pondérés. La régression logistique multivariée a été ajustée pour les covariables pertinentes afin d’identifier les facteurs sociodémographiques et les croyances associés à l’hésitation à la vaccination.

**Résultats:**

Nous avons collecté 2 202 questionnaires remplis. Un faible niveau d’éducation (lycée : rapport de cotes (OR) 1,90, intervalle de confiance (IC) à 95% 1,29, 2,81) et le fait d’avoir des enfants (OR 1,89, IC 1,39, 2,57) étaient associés à une probabilité accrue d’éprouver une hésitation à l’égard d’une dose de rappel, tandis qu’un revenu plus élevé (100 000 $–149 999 $ : OR 0,60, IC 0,39, 0,91; 150 000 $ ou plus : OR 0,49, IC 0,29, 0,82) était associé à une diminution des probabilités. Incrédulité dans l’efficacité du vaccin (contre l’infection : OR 3,69, IC 1,98, 6,90; maladie grave : OR 3,15, IC 1,69, 5,86), en désaccord avec la prise de décision du gouvernement (plutôt en désaccord : OR 2,70, IC 1,38, 5,29; fortement en désaccord : OR 4,62, IC 2,20, 9,7) et la croyance dans le sur-vaccination (OR 2,07, IC 1,53, 2,80) ont été associées à une hésitation à recevoir une dose de rappel.

**Conclusion:**

Une hésitation à l’égard du vaccin contre la COVID-19 peut se développer ou augmenter à l’égard des vaccins ultérieurs. Nos résultats indiquent des facteurs à prendre en compte lors du ciblage des populations hésitantes à la vaccination.

## Introduction

Vaccine hesitancy, described as the delay in acceptance or refusal of available vaccines (World Health Organization 2014), and a top threat to global health, hinders public health efforts to prevent and mitigate the spread and severity of the severe acute respiratory syndrome coronavirus 2 (SARS-CoV-2). As vaccines are vital tools to combat infectious diseases such as coronavirus disease 2019 (COVID-19) and protect global population health, factors influencing COVID-19 vaccine acceptance, including vaccine hesitancy, have been researched worldwide (Lazarus et al., 2021). Personal beliefs and perceptions of vaccine safety and efficacy (Olanipekun et al., 2021), trust in authorities (Olanipekun et al., 2021), consumption of misinformation (Pierri et al., 2022) and socio-demographic characteristics (Lavoie et al., 2022) are associated with COVID-19 vaccine hesitancy and describe the breadth of possible factors impacting vaccine acceptance.

The novelty of the SARS-CoV-2 further compounds the challenges of addressing COVID-19 vaccine hesitancy. While a complete COVID-19 vaccine primary series has been defined for each approved vaccine, continued viral transmission, emergence of variants of concern, and weakened immunity over time resulted in the Canadian government suggesting that the number of doses increase to include booster doses (i.e., a COVID-19 vaccine dose beyond a primary series [one or two doses, brand dependent]) in December of 2021 for adults (> 18 years) in Canada (Public Health Agency of Canada, 2021). Evidence suggests that a COVID-19 booster dose is safe, is effective (Moreira et al., 2022) and increases protection from SARS-CoV-2 infection and serious illness (Bar-On et al., 2021). However, vaccine hesitancy persists, as the number of individuals who have received a COVID-19 booster dose is lower than that of recipients of first and second doses in Canada (Government of Canada, 2021) and globally (Centers for Disease Control & Prevention, 2023; United Kingdom Government, 2023).

COVID-19 vaccination beliefs, intentions, and actions can shift over time (Lavoie et al., 2022) and reflect a continuum of vaccine acceptance, underscoring the need for continuing research into COVID-19 vaccine hesitancy, particularly as additional doses are recommended. Current research focuses primarily on vaccine hesitancy toward an initial COVID-19 vaccine, while hesitancy toward a COVID-19 booster dose is comparably underexamined (Reifferscheid et al., 2022; Wong et al., 2022). Moreover, existing research largely examines behavioural intentions (i.e., individual motivations to perform a specific behaviour (Sheeran, 2002)) to receive a booster dose prior to roll-out (Reifferscheid et al., 2022; Wong et al., 2022); there is need to examine behaviours (i.e., performing the intended behaviours (Sheeran, 2002)) toward COVID-19 booster doses post-roll-out. We deployed a cross-national population-based survey to understand Canadians’ knowledge, beliefs, and behaviours toward continued COVID-19 vaccination to support ongoing pandemic preparedness and recovery.

## Methods

We developed a cross-sectional, online, anonymous survey and contracted Leger (https://leger360.com/), a Canadian market research and analytics company, to administer the survey across Canada. The study received ethical approval from the Research Ethics Boards of Dalhousie University (#2021–5782) and the University of Calgary (#21–1241). The methods adhered to the Strengthening of Reporting Observational Studies in Epidemiology (STROBE) statement (Supplemental File 1, Table 1).

## Questionnaire design

Using standard survey methodology (Burns et al., 2008), we developed a list of 76 questions based on published survey questions on vaccine hesitancy (Khubchandani et al., 2021; Larson et al., 2015; Lazarus et al., 2021; Siddiqui et al., 2013) to capture knowledge, beliefs, and behaviours toward COVID-19 vaccines (Supplemental File 2). We included questions to examine hesitancy toward the first, second and third COVID-19 vaccine doses. For the purpose of this study, a third COVID-19 vaccine was defined as a COVID-19 booster dose. Questions were iteratively refined by the survey development team (researchers: SJM, JPL, RB, SH, DH; patient partners: KR, SL, SK). Some questions were developed de novo and reviewed by experts within the team. Based on the Strategic Advisory Group of Experts on Immunization (SAGE) Working Group definition of vaccine hesitancy (World Health Organization, 2014), we defined COVID-19 vaccine hesitancy as intentional or behavioural delay in acceptance or refusal of vaccines when available. Question types were variable response options, including 5-point Likert scales, single-response multiple choice, multiple-response checkboxes, and open-ended questions. We included an additional 26 questions to collect demographic (e.g., sex) and personal characteristics (e.g., employment). The questionnaire was administered in both English and French and pre-tested in both languages.

## Questionnaire administration

The questionnaire was distributed electronically through Leger’s Opinion (LEO) panel of approximately 400,000 adults in Canada with internet access. Participants were recruited through direct invitation from the LEO panel. Panelists were eligible if they were adults (≥ 18 years), lived in Canada, could read English or French, and were able to provide informed consent. Based on 2016 Canadian census data (Statistics Canada, 2017), we sought to recruit minimum quotas of age (18–34, 35–55, > 55 years), sex (female, male), and provincially defined regions (British Columbia, Alberta, Saskatchewan/Manitoba, Ontario, Québec, Atlantic provinces, and Territories). Respondents received Leger reward points after completing the survey; points can be redeemed for gift cards and merchandise.

## Sample size calculations

An overall sample size of 385 participants was calculated based on a standard survey sample size calculation (Enderlein, 1995) (assuming an observed proportion selecting a specific response option of 50%) with a total population size of ~ 36.3 million in Canada, and a 95% confidence interval (CI) of ± 5%. To conduct subgroup analyses (e.g., sociodemographic categories) we collected a total of 2000 surveys and calculated the associated margin of error to be ± 2.2% at a 95% CI.

## Data analysis

Descriptive statistics (i.e., frequencies, weighted percentages, means and associated 95% CIs, standard deviations (SDs)) were used to summarize respondent characteristics and survey responses. Responses were weighted by age, sex, and regional population estimates derived from 2016 Canadian census data (Statistics Canada, 2017). We tested for vaccine hesitancy between categories of sociodemographic subgroups using the Rao-Scott correction to chi-squared test for weighted categorical survey data. The demographic characteristics included: (1) sex (male; female); (2) age in years (18–29; 30–44; 45–64; 65 +); (3) education (high school diploma or less; Collège d’enseignement général et professionnel (CEGEP)/vocational college/trade; some college or university (no degree); college/university degree); (4) ethnicity (white; Asian East/South East; Asian East/Indian Caribbean; Black; Indigenous; Latin American; Middle Eastern; Mixed/Other); (5) region (British Columbia; Alberta; Saskatchewan/Manitoba; Ontario; Quebec; Atlantic Canada; Territories; (6) annual household income (< $50,000; $50,000‒$99,999; $100,000‒$149,999; ≥ $150,000); (7) Children (under the age of 18 [yes/no]); and (8) years lived in Canada (< 5 years; 5‒9 years; 10‒19 years; ≥ 20 years). Respondents unable to be categorized (i.e., responded ‘prefer not to answer’) within a specific demographic variable (e.g., years lived in Canada) were excluded from that specific sub-analysis.

Leger conducted a qualitative content analysis on open-ended survey questions (Hsieh & Shannon, 2005). To begin analysis, consultants familiarized themselves with the open-ended responses and subsequently developed emergent codes that were then coded into qualitative categories. Frequency of coding units within categories were then summated. A research team member with experience in qualitative methods (JPL) performed a quality check of the categories and coding units. The researcher and consultants were attentive to and cognizant of the influence of personal perspectives and experiences that may influence data interpretation. To recognize and improve trustworthiness of these findings (i.e., credibility, dependability, confirmability and transferability) (Nowell et al., 2017), consultants were provided space for an iterative and reflective analysis process, maintained a strong dialogue throughout the analysis (including peer debriefing), and grounded interpretations in the textual data.

Multivariable logistic regression models were used to assess the relationship between vaccine hesitancy and (1) sociodemographic variables, as well as perceptions of (2) vaccine effectiveness, (3) recommended doses, and (4) trust in government. Individuals who responded ‘somewhat agree,’ ‘no opinion,’ ‘somewhat disagree,’ or ‘strongly disagree’ on a 5-point Likert scale when asked if they would receive the next COVID-19 vaccine dose that they were due for, or if they responded ‘no’ when asked if they had ever received a COVID-19 vaccine dose, were coded as vaccine hesitant (Supplemental File 2). Individuals who responded ‘strongly agree’ on a 5-point Likert scale when asked if they would receive the next COVID-19 vaccine dose that they were due for, or if they responded ‘yes’ when asked if they had ever received a COVID-19 vaccine dose, were coded as vaccine acceptant (Supplemental File 2). ‘Prefer not to answer’ responses were excluded from analyses. A backward stepwise linear regression was used to identify possible predictors of vaccine hesitancy. At each step, variables were chosen based on a *p*-value threshold (< 0.1). The odds ratio (OR) sociodemographic reference groups were female (sex), college/university degree(s) (education), Ontario (region), < $50 K (income), < 5 years (lived in Canada), and white (ethnicity). We conducted quantitative data analyses using SAS 9.4; *p* < 0.05 indicated statistical significance.

## Results

We collected 2202 eligible questionnaires between March 16 and March 26, 2022, during which time booster doses were available to most adults in the general population of Canada (Public Health Agency of Canada, 2021). Just over half of respondents (*n* = 1186; 51.4%) were female and had completed a college or university degree (*n* = 1243, 54.4%) (Table 1). Over two thirds (*n* = 1316; 67.3%) of respondents had a household income < $100,000 and 72.7% (*n* = 1477) self-identified as white. The median age of the sample was 48 years (Interquartile Range [IQR] 29.4). A summary of respondent characteristics is presented in Table 1.

**Table 1:** Survey respondents characteristics

Characteristic	Number^a^	Weighted %
Gender (*N* = 2200)		
Women	1180	51.2
Men	1006	48.2
Non-binary/Two-spirit/Self-describe	14	0.6
Sex (*N* = 2202)		
Female	1186	51.4
Male	1016	48.6
Age (*N* = 2202)		
Mean (SD)	41.8 (0.1)	
18–29	399	17.6
30–44	639	25.9
45–64	736	35.4
65 +	428	21.1
Geographical region (*N* = 2202)		
British Columbia	290	13.4
Alberta	246	11.1
Manitoba/Saskatchewan	160	6.5
Ontario	852	38.4
Quebec	519	23.5
Atlantic^b^	127	6.9
Territories^c^	8	0.3
Ethnic origins (*N* = 2144)^d^		
Asian East/Southeast	244	10.5
Asian South/Indian Caribbean	137	4.9
Black	90	3.2
Indigenous	34	1.7
Latin American	29	0.9
Middle Eastern	40	1.4
White	1477	72.7
Mixed/Other	93	4.6
Highest education completed (*N* = 2187)		
High school or less	351	17.1
CEGEP/Vocational College/Trade	309	15.1
Some College/University (no degree)	284	13.4
College/University degree(s)	1243	54.4
Total household income (*N* = 1933)		
$0–$49,999	584	29.8
$50,000–$99,999	732	37.5
$100,000–$149,999	375	19.5
$150,000 or more	242	13.1
Federal political alignment (*N* = 2133)		
The Conservative Party	411	20.1
The Liberal Party	575	26.6
The New Democratic Party	363	17.1
The Bloc Québécois	107	5.4
The Green Party	60	3.0
Other Independent Party	47	2.4
Would not vote/Would spoil ballot/Not sure	570	25.5
Religious identity (*N* = 2110)		
Roman Catholic	584	28.3
Protestant or other Christian	464	22.2
Muslim	74	2.5
Jewish	41	2.0
Hindu	47	1.7
Sikh	17	0.6
Other	44	2.1
No religious identity	839	40.6
Dependent child(ren) under 12 years (*N* = 2202)		
Yes	453	19.2
No	1749	80.8
Dependent child(ren) 12 years and older (*N* = 2202)		
Yes	385	17.9
No	1817	82.1
Received one dose of COVID-19 vaccine (*N* = 2202)^e^		
Yes	2057	93.3
No	145	6.7
Received a second dose of COVID-19 vaccine (*N* = 2057)^f^		
Yes	2025	98.7
No	32	1.3
Received a third dose of COVID-19 vaccine (*N* = 1886)^g^		
Yes	1514	81.1
No^h^	372	18.9
Self/Family experience with COVID-19 disease (*N* = 2202)		
Yes	1057	48.1
No	1145	51.9
Lost a family member to COVID-19 disease		
Yes	174	7.2
No	2028	91.6

^a^ Frequencies are noted unless otherwise indicated. ‘Prefer not to answer’ response options are excluded from individual N reported

^b^Atlantic Canada includes the provinces of New Brunswick, Newfoundland and Labrador, Nova Scotia, and Prince Edward Island

^c^Territories include the territories of Yukon, Northwest Territories, and Nunavut

^d^ The following categories were combined: (1) Asian East (e.g., Chinese, Japanese, Korean) and Asian Southeast (e.g., Malaysian, Filipino, Vietnamese), (2) Asian South (e.g., Indian, Pakistani, Sri Lankan) and Indian-Caribbean, (3) Black-African (e.g., Ghanian, Kenyan, Somali), Black-Caribbean (e.g., Barbadian, Jamaican), Black-North American (e.g., Canadian, American) into Black; (4) Indigenous, Inuit, First Nations, and Métis into Indigenous, (5) White-European (e.g., English, Italian, Portuguese, Russian), White-North American (e.g., Canadian, American) into white

^e^ Respondents who answered, ‘Yes’ or ‘No’ to ‘Have you received at least one dose of a COVID-19 vaccine?’ are included

^f^ Respondents who answered, ‘Yes’ to ‘Have you received at least one dose of a COVID-19 vaccine?’ were asked ‘Have you received a second dose of a COVID-19 vaccine?’

^g^ Respondents who answered, ‘Yes’ to ‘Have you received a second dose of a COVID-19 vaccine?’ were asked ‘Have you received a third dose of a COVID-19 vaccine?’

^h^ Respondents who reported ‘No’ or ‘Don’t know’ in response to ‘Are you eligible for a third dose of the COVID-19 vaccine?’ (*n* = 130) and subsequently ‘Yes’ to ‘Would you like to receive a particular brand for a third dose of the COVID-19 vaccine’ are included

Abbreviations: CEGEP, Collège d’enseignement général et professionnel (a publicly funded college providing technical, academic, vocational or a mix of programs in the province of Quebec); COVID-19, coronavirus disease 2019; N, number of respondents; NWT, Northwest Territories; SD, Standard deviation

## Past and current vaccinations

Over 93% of the sample reported receiving at least one dose of a COVID-19 vaccine, of whom 99% reported receiving a second dose; 81% of respondents who received two doses reported receiving a booster dose of a COVID-19 vaccine. Just under half of respondents (48%) had personal or familial experience with COVID-19 illness, while 7% had a family member die from COVID-19 disease (Table 1).

**Fig. 1:**
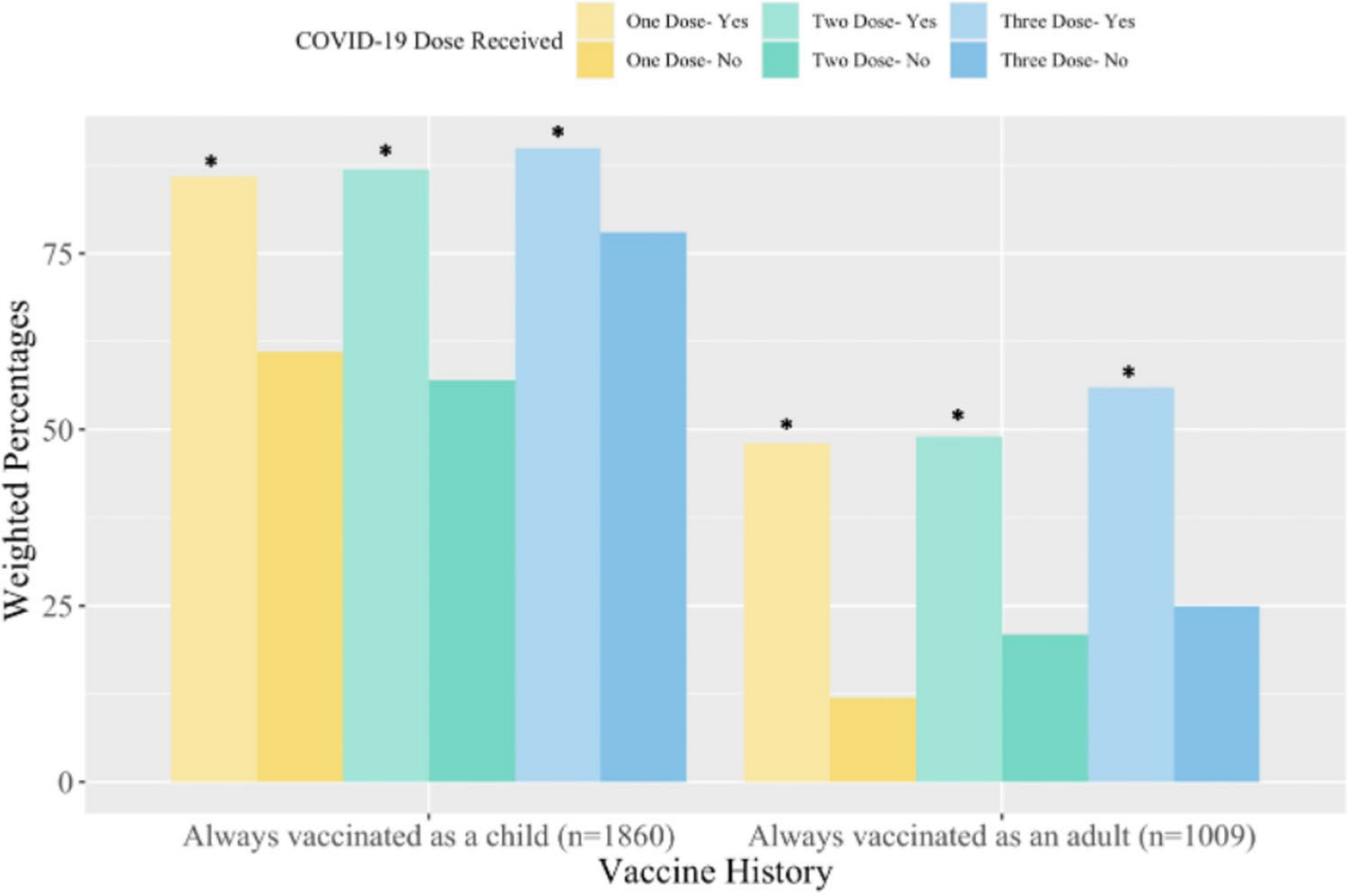
Percentage of respondents who reported always receiving scheduled vaccinations in childhood and adulthood by number of COVID-19 vaccine doses received. Respondents who reported ‘always’ to the survey questions *‘Did you receive recommended vaccinations as a child (e.g., Measles, Mumps, Rubella, or tetanus) that are not related to COVID-19?’* and *‘As an adult have you received recommended vaccinations (e.g., vaccination for seasonal influenza or tetanus boosters) that are not related to COVID-19?’* * Indicates *p* ≤ 0.01 when compared to the “No” group of the same dose

Most of our respondents (84%) indicated that they always received recommended vaccinations as a child (e.g., Measles, Mumps, Rubella), while less than half (46%) reported always receiving recommended vaccinations as an adult (e.g., seasonal influenza, tetanus boosters). A greater proportion of respondents who received a booster dose of a COVID-19 vaccine reported receiving vaccinations as a child (90%) or as an adult (56%) compared to individuals who did not receive a booster dose (78% and 25%, respectively), as was seen when stratifying by receipt of first or second dose (Fig. 1) (*p* ≤ 0.01).

## Self-perceived knowledge about and access to information regarding COVID-19 vaccines

Most (66%) respondents self-reported that they did not experience moderate to substantial difficulties accessing information on COVID-19 vaccines. Of those who experienced moderate to substantial difficulties (34%), a greater proportion of respondents who did not receive a booster dose reported experiencing difficulties (25%) compared to respondents who did receive a booster dose (11%) (*p* ≤ 0.01). Of the respondents who reported difficulties, over half (56%) noted challenges in determining the quality of the information (e.g., reliability). About two thirds of respondents self-rated their level of knowledge of COVID-19 vaccines as very good (21%) or good (43%); a greater proportion of booster dose recipients rated their COVID-19 vaccine knowledge as very good (23%) compared to respondents who had not received a booster dose (14%) (*p* ≤ 0.01). Overall, respondents rated their ability to examine health information highly (Supplemental File 3, Fig. 1).

## Beliefs regarding COVID-19 vaccines

Three quarters of respondents strongly agreed (43%) or somewhat agreed (33%) that COVID-19 is a dangerous health threat. A greater proportion of booster dose recipients (51%) strongly agreed that COVID-19 is a dangerous health threat compared to respondents without a booster dose (26%) (*p* ≤ 0.01). Less than half of respondents (43%) perceived ‘over-vaccination’ against COVID-19 possible; the proportion among respondents who did not receive a booster dose (61%) was higher compared to booster dose recipients (33%) (*p* ≤ 0.01).

Over half of respondents (60%) who received a booster dose indicated brand preference, reasoning: (1) recommendations to stay with the same brand/preferred not to mix/consistency (44%); (2) offered or available at the time (9%); (3) fewer side effects (7%); (4) preferred the brand (4%); and (5) more effective (4%) (Supplemental File 3, Fig. 2 and Table 1). Reasons for first and second dose brand preference are in Supplemental File 3, Fig. 3 and 4, respectively.

## Trust and perceptions of COVID-19 vaccine effectiveness

**Fig. 2:**
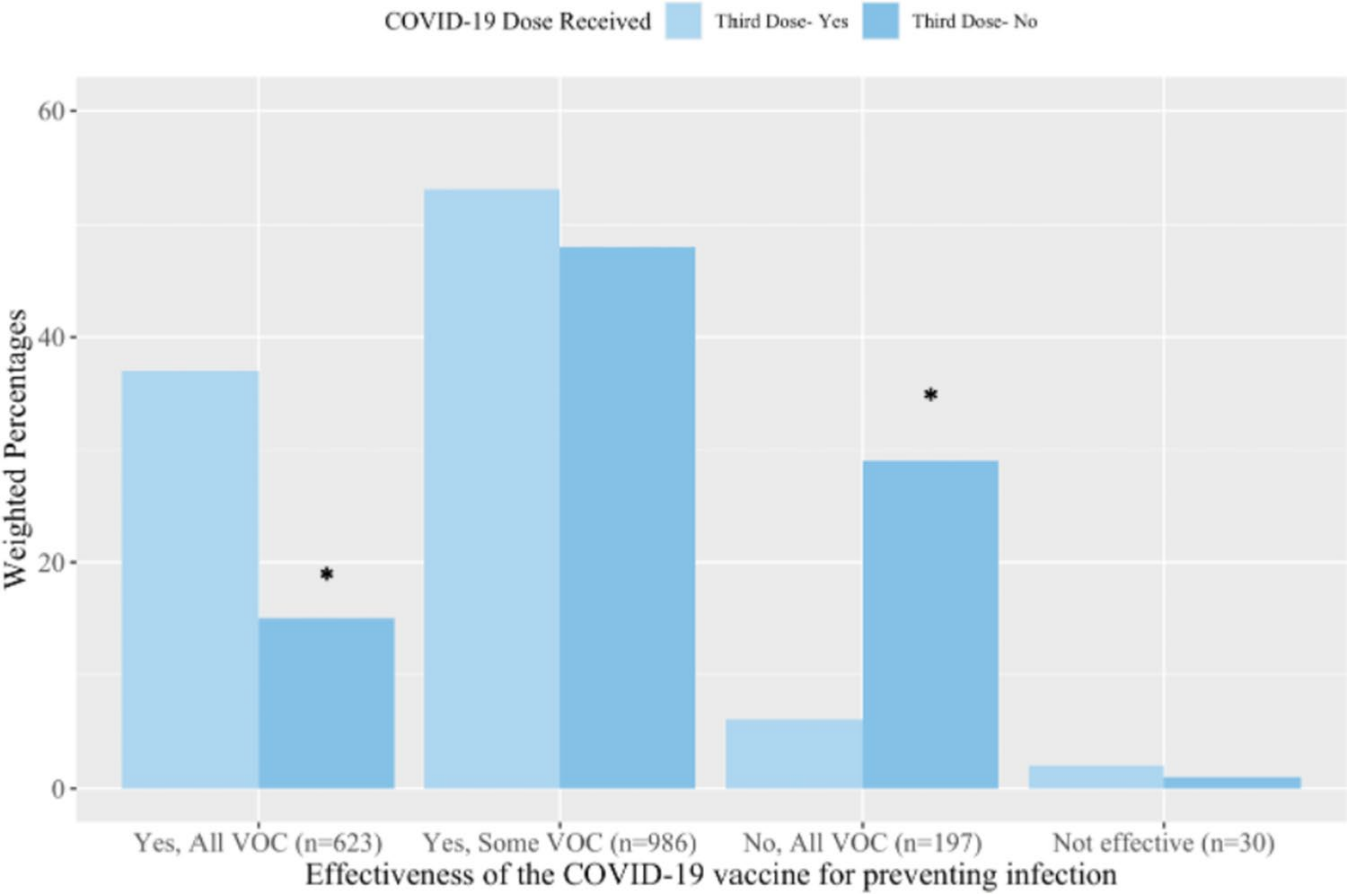
The perceived effectiveness of COVID-19 vaccines for preventing infection from variants of concern (VOC) by COVID-19 booster dose receipt. Response options applied to the survey question *‘In your opinion, are vaccines effective at preventing infection from the SARS-CoV-2 virus (the virus that causes COVID-19 disease)?* * Indicates *p* ≤ 0.01 when compared to the ‘Yes’ group of the same dose

**Fig. 3:**
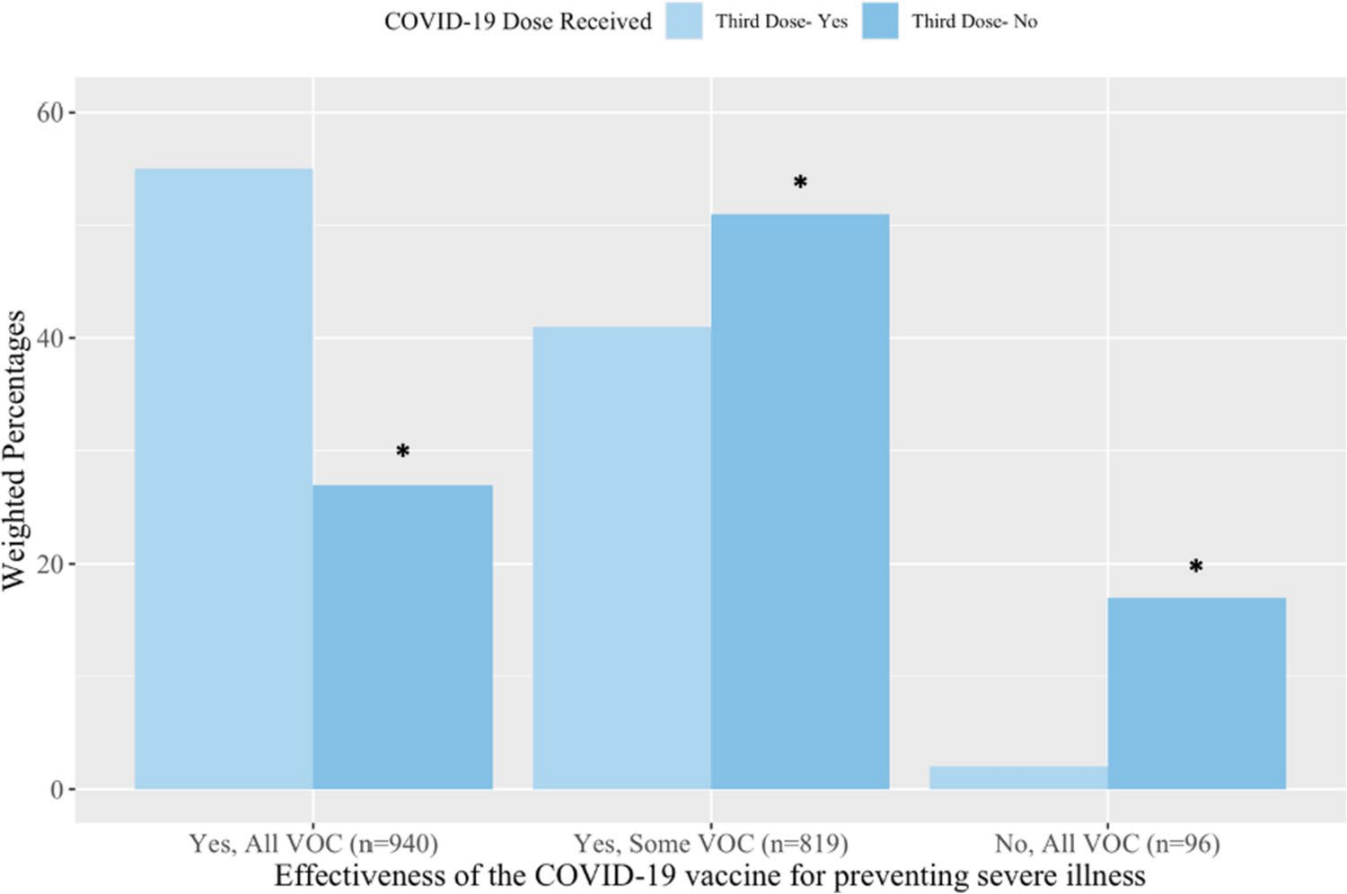
Perceived effectiveness of COVID-19 vaccines for preventing serious illness from variants of concern (VOC) by COVID-19 booster dose receipt. Response options applied to the survey question *‘In your opinion, are vaccines effective at preventing serious illness from the SARS-CoV-2 virus (the virus that causes COVID-19 disease)?.* * Indicates *p* ≤ 0.01 when compared to the ‘Yes’ group of the same dose

Just under one third of respondents (30%) believed COVID-19 vaccines were effective at preventing infection for *all* variants of concern (VOCs) of the SARS-CoV-2 virus while 49% believed COVID-19 vaccines were effective at preventing infection for *some* VOCs. A greater proportion of respondents who received a booster dose believed the effectiveness of vaccines in preventing infection against *all* VOCs (37%) than respondents without their booster dose (15%) (*p* ≤ 0.01) (Fig. 2). Respondents comparably believed the vaccines prevent serious illness from *all* VOCs (45%) or *some* VOCs (42%). A greater proportion of booster dose recipients believed that COVID-19 vaccines prevented serious illness from *all* VOCs (55%) compared to respondents without a booster dose (27%) (*p* ≤ 0.01) (Fig. 3). Comparable findings were identified when stratifying by first and second dose recipients (Supplemental File 3, Fig. 5 and 6).

A greater proportion of booster dose recipients strongly agreed and somewhat agreed (29% and 45%, respectively) with respect to their trust of federal government decision-making regarding COVID-19 vaccines, compared to respondents without a booster dose (13% and 35%, respectively) (*p* ≤ 0.01). Similarly, booster dose recipients strongly agreed and somewhat agreed (25% and 44%, respectively) with respect to their trust of provincial government decisions, compared to those without a booster dose (12% and 34%, respectively). Less than half of respondents strongly agreed (8%) and somewhat agreed (34%) with respect to their trust of the motives of pharmaceutical industries; a higher proportion of booster dose recipients somewhat agreed with respect to their trust of industry motives (38%) compared to respondents without a third dose (30%) (*p* ≤ 0.01).

## Factors associated with vaccine hesitancy toward a COVID-19 booster dose

Respondent’s hesitancy in receiving a booster dose of the COVID-19 vaccine was associated with age, income, education, having child(ren), and regional residence in Canada (Table 2). Compared to having a college or university degree, respondents with no more than a high school education had nearly twice the odds of not receiving a booster dose (OR 1.90, CI 1.29, 2.81). Participants with CEGEP, Vocational or Trades training were also more likely to experience vaccine hesitancy toward a booster dose, compared to those with a college or university degree (OR 1.86, CI 1.17, 2.96). Respondents with child(ren) < 18 years, compared to respondents without any children also had almost twice the odds of experiencing booster dose hesitancy (OR 1.89, CI 1.39, 2.57). Respondents living in Alberta compared to those in Ontario had nearly twice the odds of booster dose vaccine hesitancy (OR 1.68, CI 1.08, 2.63). A higher income was associated with decreased odds of experiencing vaccine hesitancy toward a booster dose ($100,000‒$149,999, OR 0.60, CI 0.39, 0.91; ≥ $150,000, OR 0.49, CI 0.29, 0.82). Sociodemographic variables associated with vaccine hesitancy toward a first dose of a COVID-19 vaccine also included having children, and income, however, educational attainment was not found significantly associated to first dose vaccine hesitancy (Table 3). In comparison to booster dose hesitancy, sex was found to be associated to first dose vaccine hesitancy (Table 3).

**Table 2:** Odds of experiencing booster dose vaccine hesitancy by sociodemographics

Covariate	Categories	Overall *p*-value	Strata *p*-value	OR (Adjusted)	95% CI
Age	(Continuous)	** < 0.001**		0.951	0.942, 0.961
Income	$0‒$49,999 (Reference category)	**0.020**			
	$50,000‒$99,999		0.170	0.785	0.555, 1.10
	$100,000‒$149,999		**0.017**	0.598	0.392, 0.912
	$150,000 or more		**0.007**	0.487	0.289, 0.821
Education	College/University Degree (Reference category)	**0.002**			
	High school or less		**0.001**	1.903	1.291, 2.806
	CEGEP/Vocational college/Trade		**0.009**	1.859	1.168, 2.959
	Some College or University (no degree)		0.781	0.938	0.598, 1.472
With child(ren) under the age of 18 years	No (Reference category)	** < 0.001**			
	Yes		** < 0.001**	1.889	1.389, 2.570
Region	Ontario (Reference category)	** < 0.001**			
	British Columbia		0.953	0.987	0.636, 1.531
	Alberta		**0.022**	1.683	1.077, 2.633
	Saskatchewan/Manitoba		0.846	1.063	0.573, 1.973
	Quebec		0.590	0.890	0.581,1.362
	Atlantic		0.205	1.418	0.826, 2.432
	Territories		< 0.001	< 0.001	0.000, 0.000

Logistic regression model selection was conducted from the fitted model using backward stepwise selection with elimination stopping rule set to *p* < 0.1. Responses of “Prefer not to answer” for each independent variable were excluded from the dataset. Bolded numbers are considered statistically significant results (*p* < 0.05)

**Table 3:** Odds of experiencing first dose vaccine hesitancy by sociodemographics

Covariate	Categories	Overall *p*-value	Strata *p*-value	OR (Adjusted)	95% CI
Income	$0‒$49,999 (Reference category)	**0.002**			
	$50,000‒$99,999		**0.043**	0.605	0.372, 0.984
	$100,000‒$149,999		**0.001**	0.334	0.175, 0.639
	$150,000 or more		**0.010**	0.366	0.170, 0.787
With child(ren) under the age of 18 years	No (Reference category)	** < 0.001**			
	Yes		** < 0.001**	2.318	1.504, 3.574
Sex	Female (Reference category)	**0.052**			
	Male		**0.052**	1.518	0.997, 2.312

Logistic regression model selection was conducted from the fitted model using backward stepwise selection with elimination stopping rule set to *p* < 0.1. Responses of “Prefer not to answer” for each independent variable were excluded from the dataset. Bolded numbers are considered statistically significant results (*p* < 0.05)

Respondent perceptions of vaccine effectiveness, recommended dosing, and trust in federal and provincial government decision-making were associated with booster dose vaccine hesitancy (Table 4). Those who reported that vaccines were not effective at preventing infection or serious illness to all VOCs had over three times the odds of experiencing vaccine hesitancy toward a booster dose, compared to those who felt the vaccine was effective for both (infection: OR 3.69, CI 1.98, 6.90; serious illness: OR 3.15, CI 1.69, 5.86). Respondents who perceived ‘over-vaccination’ with respect to COVID-19 possible had twice the odds of experiencing vaccine hesitancy toward a booster dose compared to respondents who did not think one could be vaccinated too many times for COVID-19 (OR 2.07, CI 1.53, 2.80). When asked whether they trusted federal government decision-making, those participants who somewhat disagreed had nearly three times greater odds of experiencing booster dose hesitancy compared to those who strongly agreed, while those who strongly disagreed had nearly five times higher odds (somewhat disagree, OR 2.70, CI 1.38, 5.29; strongly disagree, OR 4.62, CI 2.20, 9.71). Respondents who reported they were unsure or had no opinion with regard to trust in provincial government decision-making were over three times more likely to experience booster dose hesitancy (OR 3.13, CI 1.55, 6.34). Variables associated with vaccine hesitancy toward first dose of a COVID-19 vaccine are presented in Table 5.

**Table 4:** Odds of experiencing booster dose vaccine hesitancy given trust and beliefs

Covariate	Categories	Overall *p*-value	Strata *p*-value	OR (Adjusted)	95% CI
Effectiveness of vaccines at preventing infection	Yes, to all VOC (Reference category)	** < 0.001**			
	Yes, to some VOC		0.407	1.239	0.747, 2.054
	No, to no VOC		** < 0.001**	3.693	1.976, 6.900
	Other		**0.019**	4.011	1.251, 12.86
Effectiveness of vaccines at preventing serious illness	Yes, to all VOC (Reference category)	**0.002**			
	Yes, to some VOC		**0.007**	1.773	1.173, 2.679
	No, to no VOC		** < 0.001**	3.149	1.693, 5.857
	Other		0.232	2.076	0.626, 6.884
Possibility to be vaccinated too many times	No (Reference category)	** < 0.001**			
	Yes		** < 0.001**	2.070	1.533, 2.796
Trust the Canadian federal government is making decisions in best interest	Strongly agree (Reference category)	** < 0.001**			
	Somewhat agree		0.179	1.496	0.830, 2.697
	Unsure/no opinion		0.237	1.522	0.759, 3.052
	Somewhat disagree		**0.004**	2.697	1.376, 5.285
	Strongly disagree		** < 0.001**	4.624	2.202, 9.710
Trust the provincial government is making decisions in best interest	Strongly agree (Reference category)	**0.008**			
	Somewhat agree		**0.035**	1.930	1.046, 3.562
	Unsure/no opinion		**0.001**	3.131	1.547, 6.336
	Somewhat disagree		0.234	1.497	0.770, 2.910
	Strongly disagree		0.590	1.234	0.574, 2.649

Logistic regression model selection was conducted from the fitted model using backward stepwise selection with elimination stopping rule set to *p* < 0.1. Responses of “Prefer not to answer” for each independent variable were excluded from the dataset. Bolded numbers are considered statistically significant results (*p* < 0.05). VOC, Variants of concern

**Table 5:** Odds of experiencing first dose vaccine hesitancy given trust and beliefs

Covariate	Categories	Overall *p*-value	Strata *p*-value	OR (Adjusted)	95% CI
Effectiveness of vaccines in preventing infection	Yes, to all VOC (RC)	**0.020**			
	Yes, to some VOC		0.771	1.204	0.344, 4.215
	No, to no VOC		0.088	2.910	0.851, 9.956
	Other		0.561	0.462	0.034, 6.240
Effectiveness of vaccines at preventing serious illness	Yes, to all VOC (RC)	** < 0.001**			
	Yes, to some VOC		** < 0.001**	16.19	3.683, 71.20
	No, to no VOC		** < 0.001**	54.08	12.09, 241.9
	Other		** < 0.001**	49.91	6.043, 364.2
Possibility to be vaccinated too many times	No (RC)	**0.003**			
	Yes		**0.003**	2.496	1.365, 4.562
Trust the provincial government is making decisions in best interest	Strongly agree (RC)	** < 0.001**			
	Somewhat agree		0.834	1.165	0.280, 4.857
	Unsure/no opinion		0.527	1.592	0.376, 6.737
	Somewhat disagree		0.105	3.252	0.781, 13.54
	Strongly disagree		**0.003**	7.901	2.003, 31.16

Logistic regression model selection was conducted from the fitted model using backward stepwise selection with elimination stopping rule set to *p*-value < 0.1. Response option “Prefer not to answer” from each independent variable were excluded from the dataset. Bolded numbers are considered statistically significant results (< 0.05). VOC, Variants of concern; RC, Reference category

## Discussion

In our cross-national survey of over 2000 adults in Canada, we found an 18% drop between primary series and booster dose vaccine uptake, despite public health recommendations and availability of booster doses across Canada (Public Health Agency of Canada, 2021). Our study found certain demographic characteristics including age, income, education, having child(ren), and regional residence in Canada to be associated with booster dose vaccine hesitancy. Beliefs in vaccine efficacy and recommended dosing, and trust in federal and provincial government decision-making were also associated with hesitancy toward a booster dose. The data suggest that individuals may develop vaccine hesitancy toward continued vaccination for COVID-19 and these should be considered a priority population to target in current and future vaccine campaigns. Prevention and mitigation of infectious diseases through widespread vaccination uptake underpin public health and pandemic preparedness in Canada and beyond.

Current research on COVID-19 booster doses focuses primarily on behavioural *intentions* to receive a COVID-19 booster dose (Reifferscheid et al., 2022; Wong et al., 2022). Our research provides data on both intentions *and* actual behaviour toward booster doses, with 81% of primary series recipients subsequently receiving a booster dose. This uptake is high in comparison to a Canadian report from May 2022 that found 59% of adults in Canada were fully vaccinated (i.e., primary series) and had at least one additional dose (i.e., booster dose) (Government of Canada, 2022). In our study, a greater proportion of booster dose recipients reported COVID-19 as a health threat, compared to those who did not receive a booster dose. Similarly, participants who received a booster dose were more likely than those who did not to believe in the efficacy of the COVID-19 vaccine. The Health Belief Model (HBM) hypothesizes that a combination of factors influences health-related behaviour (e.g., receiving vaccination), including perceived susceptibility, severity, benefits, barriers, cues to action, and self-efficacy (Abraham & Sheeran, 2014). Our findings suggest that perceived severity of COVID-19, and perceived benefits of the booster dose, may influence Canadians’ hesitancy toward continued COVID-19 vaccinations. A recent meta-analysis conducted in Canada found that hesitancy toward a first dose of COVID-19 was associated with beliefs that COVID-19 would not affect themselves nor those around them, and that benefits of the vaccine do not outweigh the risks (Cénat et al., 2023). These findings suggest that similar factors may be associated with hesitancy toward a booster dose. More research is needed to examine whether and how these factors may shift between doses and throughout a pandemic, as one study conducted in Canada found the prevalence of vaccine hesitancy toward a first COVID-19 vaccine shifting over time throughout subsequent pandemic waves (Lavoie et al., 2022). Consistent with other recommendations (Lavoie et al., 2022), our research highlights the need for continued communication surrounding the benefits and importance of getting vaccinated through multi-dose vaccine schedules.

First dose COVID-19 vaccine hesitancy has been found associated with exposure to and consumption of misinformation (Pierri et al., 2022). Researchers have also found health literacy associated with COVID-19 vaccine acceptance, where the risk of hesitancy toward a first vaccine was higher among individuals with lower health literacy scores (Montagni et al., 2021). In our study, respondents who did not receive a booster dose reported difficulty with accessing information and assessing the quality of information related to COVID-19 vaccines. Respondents without a booster dose less frequently self-rated their COVID-19 knowledge as good compared to booster dose recipients. This finding may reflect lower health literacy among respondents without a booster dose, or an acknowledgement and acceptance of an overall lack of knowledge on a rapidly evolving body of research (e.g., VOCs, effectiveness between brands over time) (Feikin et al., 2022). Considering the association of health literacy and health disparities (Schillinger, 2021), and that the SAGE Working Group describe communication as a tool, not a determinant, toward vaccine hesitancy (MacDonald & SAGE Working Group on Vaccine Hesitancy, 2015), we suggest a need for policy makers to ensure individualized communication targeted to vulnerable sub-groups in the general population to facilitate accessible and assessable information for all members of the public.

The importance of targeting specific sub-groups in the Canadian population to promote vaccine uptake is exemplified in our examination of demographic variables associated with COVID-19 vaccine hesitancy. Similar to research that found Canadians with higher income had increased odds of accepting additional COVID-19 vaccines (Reifferscheid et al., 2022), our study found participants with higher income, compared to lower income, were associated with decreased odds of experiencing booster dose vaccine hesitancy. This same study found higher educational attainment associated with higher odds of accepting additional COVID-19 doses (Reifferscheid et al., 2022), similar to our findings of lower educational attainment associated with increased odds of experiencing booster dose hesitancy. These findings suggest future vaccine campaigns should target individuals with lower socioeconomic status to promote COVID-19 booster dose uptake. Interestingly, in our findings of vaccine hesitancy toward a first COVID-19 vaccine, we found no significant difference between educational attainment and hesitancy toward a first COVID-19 vaccine. In a study examining factors associated with first dose vaccine hesitancy and unwillingness in Canada, there were no significant differences found in hesitancy toward a COVID-19 vaccine between secondary or less and postsecondary education (Cénat et al., 2023). Additionally, sex was not consistently found to be significantly associated with COVID-19 vaccine hesitancy. Males were found more likely to experience hesitancy toward a first COVID-19 vaccine, compared to females, while sex was not found to be significantly associated with booster dose vaccine hesitancy. These findings suggest that demographic factors associated with COVID-19 vaccine hesitancy may differ between doses and future research should examine the evolution of these factors *between* doses to ensure immunization campaigns target appropriate sub-groups regarding specific doses for optimal uptake.

### Strengths and limitations

Our study benefits from several strengths, including a large cross-national sample of adults in Canada. Our survey was implemented several months after Canadian officials updated guidance and recommendations for adults in Canada to receive a booster dose (Public Health Agency of Canada, 2021); booster doses were largely available to the Canadian public at time of survey (Public Health Agency of Canada, 2021). In accordance with the SAGE Working Group definition of vaccine hesitancy (World Health Organization, 2014), the availability of the booster dose allowed for the examination of behavioural hesitancy toward booster doses, rather than solely intentions, as is found in much of the extant literature. However, this study also has limitations. Although our survey obtained a large sample of Canadians based on age, sex, and geographical location, the survey was conducted online and in English and French languages. This limited the opportunity to elicit opinions from individuals without internet access and from those who read and write in different languages. We recruited survey respondents from a pre-established panel of volunteers (LEO panel) through Leger that may have introduced selection bias. For example, individuals who join the LEO panel may have stronger views and interests to voice their opinions than the general public on topics such as COVID-19; however, Leger applies rigorous processes to recruit participants to their volunteer panel to ensure a broad sample of the Canadian population (Leger, 2022). The time frame of survey deployment is important given the evolving SARS-CoV-2 virus and subsequent vaccine recommendations. A small number of respondents noted ineligibility for a booster dose that may reflect health conditions or specific provincial regulations (e.g., recommended time after primary series or COVID-19 infection); this small number of respondents is unlikely to have impacted our findings. Our definition of a booster dose limited the inclusion of Janssen (Johnson & Johnson) vaccine recipients, and we relied on self-reported vaccine behaviours, which we were unable to verify. Further, a cross-sectional survey is limited in its ability to elicit in-depth and contextualized answers and open text boxes were not provided to obtain more detailed responses. We developed broad, neutral survey questions to collect reliable answers that were core to the research subject; however, it is possible that misinterpretations of survey questions (e.g., regarding trusting the motives of the pharmaceutical industry) occurred that were not able to be corrected at the time of survey completion. Finally, a non-negligible proportion of our sample self-reported having lost a family member due to COVID-19. This indicates a possible selection bias such that individuals who had lost family members compared to those who had not were more likely to participate in the survey. Considering the possibility that individuals who self-reported having lost a family member to COVID-19 were more likely than not to have received all available vaccinations, our data potentially underestimate the true prevalence of vaccine hesitancy.

## Conclusion

The impact of vaccine hesitancy on recommended booster doses for COVID-19 vaccines is multidimensional and associated with a variety of factors. Future vaccination campaigns should ensure accessible public health messaging on vaccine safety and efficacy and the importance of continued vaccination uptake that appropriately target sub-demographic groups with lower vaccination uptake. Further research is needed to develop a deeper understanding of individual decision-making regarding vaccination as booster doses are introduced.

## Contributions to knowledge

What does this study add to existing knowledge?

- Factors associated with vaccine hesitancy toward a first COVID-19 vaccine may differ from factors associated with hesitancy toward booster doses.
- Demographic factors including age, income, education, parental status, and region were associated with vaccine hesitancy toward a COVID-19 booster dose.
- Personal perceptions and beliefs in COVID-19 vaccine efficacy, appropriate dosing, and trust in government were associated with vaccine hesitancy toward a COVID-19 booster dose.

What are the key implications for public health interventions, practice or policy?

- Our findings demonstrate that vaccine hesitancy may develop or increase throughout continued vaccination against COVID-19; targeted public health messaging is needed to maximize uptake.

### Supplementary Information

Below is the link to the electronic supplementary material.

Supplementary file1 (DOCX 32 KB)

Supplementary file2 (DOCX 114 KB)

Supplementary file3 (DOCX 539 KB)

## Data Availability

The datasets generated and analyzed are not publicly available as we did not secure direct permission from the survey respondents to share the de-identified dataset with the general public. Requests for the data can be directed to the institutional research ethics boards overseeing the conduct of the study via the corresponding author, Dr. Jeanna Parsons Leigh.
