# Supplementary material for: Factors affecting hesitancy toward COVID-19 vaccine booster doses in Canada: a cross-national survey
Source: Can J Public Health. 2023 Nov 22;115(1):26–39. doi: 10.17269/s41997-023-00823-z (PMC10853155; doi:10.17269/s41997-023-00823-z)
Supplement: Supplementary file 2 — Supplementary file2 (DOCX 114 KB) [file 41997_2023_823_MOESM2_ESM.docx]

**Supplemental File 1**

**Drivers of Vaccine Hesitancy**

*Public Perceptions Survey*

**Dans quelle langue souhaitez-vous répondre à ce sondage? / In what language would you like to complete this survey?**

**O** Français

**O** English

[After language selection, survey begins with implied consent in appropriate language then S1]

**SCREENING QUESTIONS FOR QUOTA MANAGEMENT**

**S1. How old are you?**

RESP_AGE

Single response

**[THANK AND TERMINATE IF UNDER 18]**

**S2. What gender do you most identify with?**

**O** Woman

**O** Man

**O** Non-binary

**O** Two-spirited

**O** Prefer to self-describe: __________

**O** Prefer not to answer

**S3. Please indicate your sex?** *As indicated by Statistics Canada, transgender, transsexual, and intersex Canadians should indicate the sex (male or female) with which they most associate themselves.*

**O** Female

**O** Male

**O** Prefer not to answer

**S4. What province or territory do you live in?**

**O** British Columbia

**O** Alberta

**O** Saskatchewan

**O** Manitoba

**O** Ontario

**O** Quebec

**O** Newfoundland and Labrador

**O** Prince Edward Island

**O** New Brunswick

**O** Nova Scotia

**O** Yukon

**O** Northwest Territories

**O** Nunavut

**MAIN QUESTIONNAIRE BODY**

**To control order response bias from subjective assessment, the direction of all ordinal scales should be allocated randomly. Back-scrolling will not be allowed.**

**1. COVID-19 is a dangerous health threat in Canada.**

**O** Strongly agree

**O** Somewhat agree

**O** Unsure/no opinion

**O** Somewhat disagree

**O** Strongly disagree

**2. Please rate your level of knowledge about COVID-19 vaccines.**

**O** Very good

**O** Good

**O** Average

**O** Poor

**O** Very poor

**3. Have you ever actively looked for information about COVID-19 vaccines?**

**O** Yes **[SKIP TO Q5]**

**O** No

**O** I don’t know or remember

**4. Where would you like to get information about COVID-19 vaccines?**

*Please select all that apply.*

**[RESPONDENTS TO THIS QUESTION WILL SKIP TO Q10]**

**O** A family member

**O** A friend or co-worker

**O** A healthcare provider (e.g., doctor, nurse) in a hospital

**O** A healthcare provider (e.g., doctor, nurse) in a community clinic

**O** A complementary health practitioner (e.g., naturopathic doctor)

**O** A newspaper or magazine (ad or article)

**O** A scientific journal article(s)

**O** The television (medical series, medical news, interviews with healthcare providers)

**O** The radio

**O** The internet (ad or website)

**O** A social media platform such as Facebook, Twitter, Instagram, etc.

**O** Application downloaded to my cellphone or tablet

**O** School or an education session (e.g., lecture, workshop)

**O** Other (please specify) ________________

**O** I don’t know how I would like to get information about COVID-19 vaccines.

**O** I don’t want to get information about COVID-19 vaccines

**5. Where have you looked for information about COVID-19 vaccines?**

*Please select all that apply.*

**O** A family member

**O** A friend or co-worker

**O** A healthcare provider (e.g., doctor, nurse) in a hospital

**O** A healthcare provider (e.g., doctor, nurse) in a community clinic

**O** A complementary health practitioner (e.g., naturopathic doctor)

**O** A newspaper or magazine (ad or article)

**O** A scientific journal article(s)

**O** The television (medical series, medical news, interviews with healthcare providers)

**O** The radio

**O** The internet (ad or website) **[DISPLAY Q7 IF NOT SELECTED]**

**O** A social media platform such as Facebook, Twitter, Instagram, etc.

**O** Application downloaded to my cellphone or tablet

**O** School or an education session (e.g., lecture, workshop)

**O** Other (please specify) ________________

**O** I don’t know or remember where I looked for information about COVID-19 vaccines

**6. Which of the following reasons MOST influenced where you looked for information about COVID-19 vaccines?** *Please select one option only.*

**O** Recommendation from someone else

**O** Ease of access to information

**O** User friendly format

**O** Reliability of information

**O** Trust in information source

**O** Low cost

**O** Confidentiality of information

**O** Other (please specify) ___________

**O** I don’t know or remember

**7. What was your main reason for NOT using the internet to look for information about COVID-19 vaccines?** *Please select one option only.*

**[RESPONDENTS TO THIS QUESTION DID NOT SELECT INTERNET IN Q5]**

**O** I think that the information provided on the internet is not reliable

**O** I think that the information provided on the internet cannot be trusted

**O** I find it difficult to find the information I am looking for using online sources

**O** I did not have reliable access to the internet

**O** Other (please name the reasons(s)): _____

**8. Did you experience any difficulties getting the information you wanted or needed about COVID-19?**

**O** Yes

**O** No **[SKIP TO Q10]**

**O** I don’t know or remember

**9. Please select the difficulties that you experienced in getting the information you wanted or needed about COVID-19.** *Please select all that apply.*

**O** Information was not explained in plain language

**O** Lack of information on the topic I was searching

**O** Lack of information available on demand (when I needed it)

**O** Lack of time to access information in general

**O** Difficulty in determining the quality of the information (i.e., is the information is reliable)

**O** Other (please specify) ________________

**O** I don’t know or remember

***For the following five questions, please select your level of agreement.***

|  | Completely Disagree | Somewhat Disagree | Somewhat Agree | Completely Agree |
| --- | --- | --- | --- | --- |
| **10. I compare health information from different sources** | **O** | **O** | **O** | **O** |
| **11. When I hear about or read about new health information I verify if it is true or not** | **O** | **O** | **O** | **O** |
| **12. I decide what health information is best for me** | **O** | **O** | **O** | **O** |
| **13. I can identify if health information is relevant to me or not** | **O** | **O** | **O** | **O** |
| **14. I ask a health professional about the quality of information I find** | **O** | **O** | **O** | **O** |

**15. Briefly describe (1-3 sentences) in your own words what is vaccine hesitancy?**

**[RESPONSE REQUIRED]**

|  |
| --- |

**16. Did you receive recommended vaccinations as a child (e.g., Measles, Mumps, Rubella, or tetanus) that are not related to COVID-19?**

**O** Always

**O** Sometimes

**O** Never

**O** Unsure

**O** Prefer not to answer

**17. As an adult have you received recommended vaccinations (e.g., vaccination for seasonal influenza or tetanus boosters) that are not related to COVID-19?**

**O** Always

**O** Sometimes

**O** Never

**O** Not applicable

**O** Prefer not to answer

**18. Have you received at least one dose of a COVID-19 vaccine?**

**O** Yes

**O** No **[SKIP TO Q20]**

**19. Did you want to receive a particular brand for a first dose of the COVID-19 vaccine?**

**O** Yes **[SKIP TO Q24]**

**O** No **[SKIP TO Q26]**

**20. I will receive a COVID-19 vaccine.**

**O** Strongly agree

**O** Somewhat agree

**O** Unsure/no opinion

**O** Somewhat disagree

**O** Strongly disagree

**21. Briefly describe (1-3 sentences) in your own words why you did not receive a first dose of the COVID-19 vaccine?**

**[RESPONSE NOT REQUIRED]**

|  |
| --- |

**22. Would you like to receive a particular brand for a first dose of the COVID-19 vaccine?**

**O** Yes

**O** No, I do not have a brand preference **[SKIP TO Q48]**

**O** No, I will not be getting a COVID-19 vaccine **[SKIP TO Q48]**

**23. Which COVID-19 vaccine would you like to receive for a first dose?**

*One response allowed.*

**[RESPONDENTS TO THIS QUESTION WILL SKIP TO Q25]**

**O** Pfizer-BioNTech Comirnaty (mRNA)

**O** Moderna Spikevax (mRNA)

**O** I am comfortable with either of the mRNA vaccines listed above

**O** I would be more comfortable with a traditional vaccine format (e.g., protein)

**24. Which COVID-19 vaccine did you receive for a first dose?**

*One response allowed.*

**O** Pfizer-BioNTech Comirnaty

**O** Moderna Spikevax

**O** AstraZeneca Vaxzevria

**O** Janssen (Johnson & Johnson)

**25. Briefly describe (1-3 sentences) in your own words the reason(s) behind your vaccine brand preference for your first dose.**

**[RESPONSE NOT REQUIRED]**

**[RESPONDENTS TO THIS QUESTION WHO ANSWERED ‘NO’ TO Q18 WILL SKIP TO Q42 AFTER ANSWERING; RESPONDENTS WHO ANSWERED ‘YES’ TO Q18 WILL PROCEED]**

|  |
| --- |

**26. Have you received a second dose of a COVID-19 vaccine?**

**O** Yes

**O** No **[SKIP TO Q28]**

**27. Did you want to receive a particular brand for a second dose of the COVID-19 vaccine?**

**O** Yes **[SKIP TO Q32]**

**O** No **[SKIP TO Q34]**

**28. I will receive a second COVID-19 vaccine.**

**O** Strongly agree

**O** Somewhat agree

**O** Unsure/no opinion

**O** Somewhat disagree

**O** Strongly disagree

**29. Briefly describe (1-3 sentences) in your own words why you did not receive a second dose of the COVID-19 vaccine?**

**[RESPONSE NOT REQUIRED]**

|  |
| --- |

**30. Would you like to receive a particular brand for a second dose of the COVID-19 vaccine?**

**O** Yes

**O** No, I do not have a brand preference **[SKIP TO Q48]**

**O** No, I will not be getting a COVID-19 vaccine **[SKIP TO Q48]**

**31. Which brand of COVID-19 vaccine would you like to receive for a second dose?**

*One response allowed.*

**[RESPONDENTS TO THIS QUESTION WILL SKIP TO Q33]**

**O** Pfizer-BioNTech Comirnaty (mRNA)

**O** Moderna Spikevax (mRNA)

**O** I am comfortable with either of the mRNA vaccines listed above

**O** I would be more comfortable with a traditional vaccine format (e.g., protein)

**32. Which brand of COVID-19 vaccine did you receive for a second dose?**

*One response allowed.*

**O** Pfizer-BioNTech Comirnaty

**O** Moderna Spikevax

**O** AstraZeneca Vaxzevria

**O** Janssen (Johnson & Johnson)

**33. Briefly describe (1-3 sentences) in your own words the reason(s) behind your vaccine brand preference for your second dose.**

**[RESPONSE NOT REQUIRED]**

**[RESPONDENTS TO THIS QUESTION WHO ANSWERED ‘NO’ TO Q26 WILL SKIP TO Q42 AFTER ANSWERING; RESPONDENTS WHO ANSWERED ‘YES’ TO Q26 PROCEED]**

|  |
| --- |

**34. Are you eligible for a third dose of the COVID-19 vaccine?**

**O** Yes **[SKIP TO Q40]**

**O** No

**O** Don’t know

**35. I will receive a third COVID-19 vaccine when I am eligible.**

**O** Strongly agree

**O** Somewhat agree

**O** Unsure/no opinion

**O** Somewhat disagree

**O** Strongly disagree

**36. Would you like to receive a particular brand for a third dose of the COVID-19 vaccine?**

**O** Yes **[SKIP TO Q38]**

**O** No, I do not have a brand preference **[SKIP TO Q48]**

**O** No, I will not be getting a COVID-19 vaccine

**37. Briefly describe (1-3 sentences) in your own words why you do not plan to receive a third dose of the COVID-19 vaccine?**

**[RESPONSE NOT REQUIRED; RESPONDENTS TO THIS QUESTION WILL SKIP TO Q48]**

|  |
| --- |

**38. Which brand of COVID-19 vaccine would you like to receive for a third dose?**

*One response allowed.*

**O** Pfizer-BioNTech Comirnaty (mRNA)

**O** Moderna Spikevax (mRNA)

**O** I am comfortable with either of the mRNA vaccines listed above

**O** I would be more comfortable with a traditional vaccine format (e.g., protein)

**39. Briefly describe (1-3 sentences) in your own words the reason(s) behind your vaccine brand preference for your third dose.**

**[RESPONSE NOT REQUIRED]**

|  |
| --- |

**40. Have you received a third dose of a COVID-19 vaccine?**

**O** Yes

**O** No **[SKIP TO Q42]**

**41. Did you want to receive a particular brand for a third dose of the COVID-19 vaccine?**

**O** Yes **[SKIP TO Q46]**

**O** No **[SKIP TO Q48]**

**42. I will receive a third COVID-19 vaccine.**

**O** Strongly agree

**O** Somewhat agree

**O** Unsure/no opinion

**O** Somewhat disagree

**O** Strongly disagree

**43. Briefly describe (1-3 sentences) in your own words why you did not receive a third dose of the COVID-19 vaccine?**

**[RESPONSE NOT REQUIRED]**

|  |
| --- |

**44. Would you like to receive a particular brand for a third dose of the COVID-19 vaccine?**

**O** Yes

**O** No, I do not have a brand preference **[SKIP TO Q48]**

**O** No, I will not be getting a COVID-19 vaccine **[SKIP TO Q48]**

**45. Which brand of COVID-19 vaccine would you like to receive for a third dose?**

*One response allowed.*

**[RESPONDENTS TO THIS QUESTION WILL SKIP TO Q48]**

**O** Pfizer-BioNTech Comirnaty (mRNA)

**O** Moderna Spikevax (mRNA)

**O** I am comfortable with either of the mRNA vaccines listed above

**O** I would be more comfortable with a traditional vaccine format (e.g., protein)

**46. Which brand of COVID-19 vaccine did you receive for a third dose?**

*One response allowed.*

**O** Pfizer-BioNTech Comirnaty

**O** Moderna Spikevax

**O** AstraZeneca Vaxzevria

**O** Janssen (Johnson & Johnson)

**47. Briefly describe (1-3 sentences) in your own words the reason(s) behind your vaccine brand preference for your third dose.**

**[RESPONSE NOT REQUIRED]**

|  |
| --- |

**48. In your opinion, are vaccines effective at preventing infection from the SARS-CoV-2 virus (the virus that causes COVID-19 disease)?**

**O** Yes, to all variants of concern

**O** Yes, to some variants of concern

**O** No, to no variants of concern

**O** Other **[PLEASE DESCRIBE – OPEN TEXT BOX FOR RESPONSE]**

**49. In your opinion, are vaccines effective at preventing serious illness from the SARS-CoV-2 virus (the virus that causes COVID-19 disease)?**

**O** Yes, to all variants of concern

**O** Yes, to some variants of concern

**O** No, to no variants of concern

**O** Other **[PLEASE DESCRIBE – OPEN TEXT BOX FOR RESPONSE**

**50. Do you think it is possible to be vaccinated too many times for the SARS-CoV-2 virus (the virus that causes COVID-19 disease)?**

**O** Yes

**O** No **[SKIP TO Q52]**

**51. Briefly describe (1-3 sentences) in your own words why you think it is possible to be vaccinated too many times for the SARS-CoV-2 virus (the virus that causes COVID-19 disease)?**

**[RESPONSE NOT REQUIRED]**

|  |
| --- |

**52. Do you have any dependent child(ren) under the age of 12?**

**O** Yes

**O** No **[SKIP TO Q58]**

**53. Have your dependent child(ren) under the age of 12 received recommended vaccinations (e.g., Measles, Mumps, Rubella, or tetanus) that are not associated with COVID-19?**

**O** Always

**O** Sometimes

**O** Never

**O** Prefer not to answer

**54. Do you or did you want your dependent child(ren) under the age of 12 to receive a particular brand for one or more doses of their COVID-19 vaccine?**

**O** Yes **[SKIP TO Q56]**

**O** No, I do not have a brand preference **[SKIP TO Q58]**

**O** No, my dependent child would not get a COVID-19 vaccine

**55. Briefly describe (1-3 sentences) in your own words why your dependent child(ren) under the age of 12 will not or did not receive any dose of the COVID-19 vaccine?**

**[RESPONSE NOT REQUIRED]**

**[RESPONDENTS TO THIS QUESTION WILL SKIP TO Q58]**

|  |
| --- |

**56. Which brand of COVID-19 vaccine do you or did you want your dependent child(ren) under the age of 12 to receive?**

*One response allowed.*

**O** Pfizer-BioNTech Comirnaty (mRNA)

**O** Moderna Spikevax (mRNA)

**O** I am comfortable with either of the mRNA vaccines listed above

**O** I would be more comfortable with a traditional vaccine format (e.g., protein)

**57. Briefly describe (1-3 sentences) in your own words the reason(s) behind your vaccine brand preference for your dependent child(ren) under the age of 12.**

**[RESPONSE NOT REQUIRED]**

|  |
| --- |

**58. Do you have any dependent children 12 years of age or older?**

**O** Yes

**O** No **[SKIP TO Q69]**

**59. Have your dependent child(ren) 12 years of age or older received recommended vaccinations (e.g., Measles, Mumps, Rubella, or tetanus) that are not associated with COVID-19?**

**O** Always

**O** Sometimes

**O** Never

**O** Prefer not to answer

**60. Has/have your dependent child(ren) 12 years of age or older received one or more doses of the COVID-19 vaccine?**

**O** Yes **[SKIP TO Q65]**

**O** No

**61. My dependent child(ren) 12 years of age or older will receive one or more doses of the COVID-19 vaccine**

**O** Strongly agree

**O** Somewhat agree

**O** Unsure/no opinion

**O** Somewhat disagree

**O** Strongly disagree

**62. Would you like your dependent child(ren) 12 years of age or older to receive a particular brand for a first dose of the COVID-19 vaccine?**

**O** Yes **[SKIP TO Q64]**

**O** No, I do not have a brand preference **[SKIP TO Q69]**

**O** No, my dependent child(ren) 12 years of age or older will not be getting a COVID-19 vaccine

**63. Briefly describe (1-3 sentences) in your own words why your dependent child(ren) 12 years of have or older has/have not received any dose of the COVID-19 vaccine?**

**[RESPONSE NOT REQUIRED]**

**[RESPONDENTS TO THIS QUESTION WILL SKIP TO Q69]**

|  |
| --- |

**64. Which COVID-19 vaccine would you like your dependent child(ren) 12 years of age or older to receive for a first dose?**

*One response allowed.*

**[RESPONDENTS TO THIS QUESTION WILL SKIP TO Q67]**

**O** Pfizer-BioNTech Comirnaty (mRNA)

**O** Moderna Spikevax (mRNA)

**O** I am comfortable with either of the mRNA vaccines listed above

**O** I would be more comfortable with a traditional vaccine format (e.g., protein)

**65. Did you want your dependent child(ren) 12 years of age or older to receive a particular brand for one or more doses of their COVID-19 vaccine?**

**O** Yes

**O** No **[SKIP TO Q68]**

**66. Which COVID-19 vaccine did you want your dependent child(ren) 12 years of age or older to receive?**

*One response allowed.*

**O** Pfizer-BioNTech Comirnaty

**O** Moderna Spikevax

**67. Briefly describe (1-3 sentences) in your own words the reason(s) behind your vaccine brand preference for your dependent child(ren) 12 years of age or older.**

**[RESPONSE NOT REQUIRED]**

|  |
| --- |

**68. Briefly describe (1-3 sentences) in your own words why has/have your dependent child(ren) 12 years of age or older received any dose of the COVID-19 vaccine?**

**[RESPONSE NOT REQUIRED]**

|  |
| --- |

**69. Did you or someone in your family (immediate or extended) become ill with the COVID-19 disease that was diagnosed with a positive test for SARS-CoV-2 infection or medical confirmation?**

**O** Yes

**O** No

**70. Were you or someone in your family (immediate or extended) hospitalized for COVID-19 disease?**

**O** Yes

**O** No

**71. Have you lost a family member (both in household and outside of household) to COVID-19 disease?**

**O** Yes

**O** No

**72. Are you one of the following?**

**O** Physician

**O** Nurse

**O** Community Health Worker

**O** Other healthcare worker

**O** None of the above **[SKIP TO Q74]**

**73. Do you have direct in-person contact with patients on a daily basis?**

**O** Yes

**O** No

**74. I trust the motives of pharmaceutical industries.**

**O** Strongly agree

**O** Somewhat agree

**O** Unsure/no opinion

**O** Somewhat disagree

**O** Strongly disagree

**75. I trust that the Canadian federal government is making decisions in my best interest with respect to what vaccines are provided, and to whom, during the COVID-19 pandemic.**

**O** Strongly agree

**O** Somewhat agree

**O** Unsure/no opinion

**O** Somewhat disagree

**O** Strongly disagree

**76. I trust that my provincial government is making decisions in my best interest with respect to what vaccines are provided, and to whom, during the COVID-19 pandemic.**

**O** Strongly agree

**O** Somewhat agree

**O** Unsure/no opinion

**O** Somewhat disagree

**O** Strongly disagree

**DEMOGRAPHICS**

**[READ SCREEN FOR ALL]**

**We would now like to ask you some demographic questions to help categorize the responses. As a reminder, your answers will only be used in grouped data analysis – we will not be able to identify you.**

**D1. What is your current marital status?**

**O** Single, never married

**O** In a relationship, but not living together

**O** Living with partner

**O** Married

**O** Separated or divorced (in process or finalized)

**O** Widowed

**O** Prefer not to answer

**D2. What is the size of the town or city you live in?**

**O** An unincorporated area (e.g. township, village – up to 1,000 people)

**O** Small town or village (up to 5,000 people)

**O** Small city (up to 10,000 people)

**O** Medium sized city (over 10,000 people up to 100,000 people)

**O** Large city (over 100,000 people up to 1,000,000 people)

**O** Large metropolitan area (over 1,000,000 people)

**O** Don’t know

**O** Prefer not to answer

**D3. How many people live in your household, including yourself?**

*(Please include all other family members and / or roommates living in the same house)*

___ person / people [verification: integer]

**O** Prefer not to answer

**D4. How many children (under the age of 18) live in your household?** Please do not include children who are away at school. *(If you do not have any children in a specific age range, please enter ‘0’)*

**O** I do not have any children under the age of 18 years living at home

___ infants(s) (younger than 1 year1) [verification: integer]

___ toddler(s) (1 year to younger than 3 years) [verification: integer]

___ preschooler(s) (3 years to younger than 5 years) [verification: integer]

___ middle child(ren) (5 years to younger than 12 years) [verification: integer]

___ young teen(s) (12 years to younger than 16 years) [verification: integer]

___ teenager(s) (16 years to younger than 20 years) [verification: integer]

**O** Prefer not to answer

**D5. What is your current employment status? Please select all that apply.**

**O** Employed (working full-time hours)

**O** Employed (working part-time/casual hours)

**O** Self-employed (working full-time hours)

**O** Self-employed (working part-time hours)

**O** Retired

**O** Student (full or part-time)

**O** Full time parent or homemaker

**O** Currently unemployed or unable to work for any reason (including laid off)

**O** Prefer not to answer

**D6. Are you able to work from home?**

**O** Yes, full-time

**O** Yes, part-time

**O** No

**O** Prefer not to answer

**[ASK D7 IF D5 = “Currently unemployed or unable to work for any reason (including laid off)”]**

**D7.** **Is your current unemployment a direct result of the COVID-19 pandemic (e.g. laid off by a company, ordered to discontinue service from the government)?**

**O** Yes

**O** No

**O** Unsure

**O** Prefer not to answer

**[ASK D8 IF D5 = “Employed (working full-time hours)”, “Employed (working part-time/casual hours”, “Self-employed (working full-time hours)”, “Self-employed (working part-time hours)”, or “Currently unemployed or unable to work for any reason (including laid off)”]**

**D8. What is the MAIN employment sector that you work in? Examples are included. Please select the most appropriate categories.**

**O** Animal Careers
**O** Aviation
**O** Arts
**O** Business
**O** Education
**O** Law Enforcement
**O** Media
**O** Medical/Health

**O** Military Careers
**O** Service Industry

**O** Science, Technology, Engineering, and Math (STEM) Careers

**O** Other (please specify): ________________

**O** Prefer not to answer

**D9. Has the government identified your occupation as an essential service?**

**O** Yes

**O** No

**O** Unsure

**O** Prefer not to answer

**D10. What was your total overall household income in 2020 before taxes?**

**O** $0

**O** $1 to $9,999

**O** $10,000 to $24,999

**O** $25,000 to $49,999

**O** $50,000 to $74,999

**O** $75,000 to $99,999

**O** $100,000 to $149,999

**O** $150,000 to $249,999

**O** $250,000 or more

**O** Don’t know

**O** Prefer not to answer

**D11.** Which ethnic, racial, or cultural group do you most closely self-identify with? Note that the examples provided are non-exhaustive and are meant to be a guide to help you respond to the question.

*Please select all that apply.*

**O** Asian - East (e.g., Chinese, Japanese, Korean)

**O** Asian - South (e.g., Indian, Pakistani, Sri Lankan)

**O** Asian - South East (e.g., Malaysian, Filipino, Vietnamese)

**O** Black - African (e.g., Ghanaian, Kenyan, Somali)

**O** Black - Caribbean (e.g., Barbadian, Jamaican)

**O** Black – North American (e.g., Canadian, American)

**O** First Nations

**O** Indian - Caribbean

**O** Indigenous

**O** Inuit

**O** Latin American (e.g., Argentinean, Chilean, Salvadoran)
**O** Métis

**O** Middle Eastern (e.g., Egyptian, Iranian, Lebanese)

**O** White - North American (e.g., Canadian, American)

**O** White - European (e.g., English, Italian, Portuguese, Russian)

**O** Mixed heritage (please specify) ________________________________________

**O** Other racial or ethnic group (please specify) ______________________________

**O** Uncertain

**O** Prefer not to answer

**D12.** Which of the following best describes your religious/faith identity?

*Please select one response only.*

**O** Roman Catholic

**O** Protestant or other Christian

**O** Muslim

**O** Jewish

**O** Hindu

**O** Sikh

**O** Other (please specify) _____________________

**O** No Religious Identity

**O** Don't know/Prefer not to answer

**D13. Thinking of how you feel right now, if a FEDERAL election were held tomorrow, which of the following parties' candidates would you, yourself, be most likely to support?**

*Please select one response only.*

**[RANDOMIZE CODES 1-5]**

**O** The Conservative Party
**O** The Liberal Party
**O** The New Democratic Party (NDP)
**O [QUÉBEC ONLY]** The Bloc Québécois (BQ)

**O** The Green Party
**O [SHOW FOURTH TO LAST]** Some other independent party

**O [SHOW THIRD TO LAST]** Would not vote/None/Would spoil ballot)
**O [SHOW SECOND TO LAST]** Don’t know/not sure

**O [SHOW LAST]** Prefer not to answer

**[IF DON'T KNOW OR NOT SURE IN D13, ASK D13b, ELSE SKIP TO QD14]**

**D13b. Well, which party would you say you would lean towards?**

*Please select one response only.*

**[SAME ORDER AS QD12]**

**O** The Conservative Party
**O** The Liberal Party
**O** The New Democratic Party (NDP)
**O** The Green Party

**O [QUÉBEC ONLY]** The Bloc Québécois (BQ)
**O [SHOW THIRD TO LAST]** Some other independent party

**O [SHOW SECOND TO LAST]** Don’t know/not sure

**O [SHOW LAST]** Prefer not to answer

**D14. What is the highest level of education have you completed?**

**O** Less than a high school diploma

**O** High school diploma

**O** Collège d'enseignement general et professionnel (CEGEP)

**O** Vocational college

**O** Trade certification

**O** Some college (no degree)

**O** College degree

**O** Some university (no degree)

**O** Undergraduate degree (Bachelor’s)

**O** Graduate degree (Masters or Doctorate)

**O** Professional degree (MD, JD, DDS, etc.)

**O** Prefer not to answer

**D15. What kind of residence do you live in?**

**O** Detached home

**O** Semi-detached home (e.g. duplex, townhouse)

**O** Apartment or condominium

**O** School residence or dormitories

**O** Assisted living or care home

**O** Other communal housing (e.g. transition house, shelter)

**O** Other (please specify) __________________________

**O** Prefer not to answer

**D16.** **How long have you lived in Canada?**

**O** Less than 1 year

**O** 1 year up to 5 years

**O** 5 years up to 10 years

**O** 10 years up to 20 years

**O** 20 years or more

**O** I don’t know

**O** I prefer not to answer

**D17.** **How many generations INCLUDING yourself have lived in Canada?**

**O** 1 (i.e., just yourself)

**O** 2 (e.g., yourself and your parents)

**O** 3 (e.g., yourself, your parents, and your grandparents)

**O** 4+ (e.g., yourself, your parents, grandparents, and great grandparents)

**O** I don’t know

**O** I prefer not to answer

**D18. Are you currently diagnosed with any of the following health conditions?**

*Please select all that apply.* *Please do not select any conditions that you no longer have.*

**O** Autoimmune disease (e.g. lupus, psoriasis, rheumatoid arthritis, Crohn’s disease, etc.)

**O** Cancer

**O** Diabetes (Type 1 or Type 2)

**O** Cardiovascular disease (e.g. arrhythmias, coronary artery diseases, hypertension)

**O** Obesity (body mass index ≥30 kg/m^2^)

**O** Other chronic diseases (e.g. high cholesterol, kidney disease, osteoarthritis, neuromuscular, etc.)

**O** Other (please specify) ________________

**O** I am not currently diagnosed with any of these health conditions

**O** Prefer not to answer

**D19. Is anyone in your immediate family (both in and out of household) currently diagnosed with any of the following health conditions?**

*Please select all that apply.* *Please do not select any conditions that they no longer have.*

**O** Autoimmune disease (e.g. lupus, psoriasis, rheumatoid arthritis, Crohn’s disease, etc.)

**O** Cancer

**O** Diabetes (Type 1 or Type 2)

**O** Cardiovascular disease (e.g. arrhythmias, coronary artery diseases, hypertension)

**O** Obesity (body mass index ≥30 kg/m^2^)

**O** Other chronic diseases (e.g. high cholesterol, kidney disease, osteoarthritis, neuromuscular, etc.)

**O** Other (please specify) ________________

**O** I have no immediate family diagnosed with any of these health conditions

**O** Prefer not to answer

**D20. Are you currently pregnant?**

**O** Yes

**O** No

**O** Prefer not to answer

**D21. Are you a current or former smoking?**

**O** Yes

**O** No

**O** Prefer not to answer

**[READ SCREEN]**

From time to time, our research team asks people if they would be willing to be contacted to participate in follow up research with us that is related to the survey topic. This may involve participating in interviews or focus groups, either in person or online. In focus groups, we gather a group of people to talk about issues of interest to our research topic. There may also be an incentive like a gift card offered for participating.

If you are interested in being contacted about future studies, we will ask you to provide your first name and contact information. By agreeing to be contacted, you are consenting for a member of our research team to email you with details about other studies we are conducting. Your name will be added to a list of contacts for future participation for up to 1 year. This does not guarantee that you will be contacted. If you are contacted about a study, you are free to choose to participate or not participate. The contact list will be saved in a password protected file on an encrypted server at the University of Calgary. Only members of our research team will have access to this list. You may withdraw your consent to be contacted by contacting the study team at C3ResNetwork@ucalgary.ca. Your contact information will not be linked at all to your current survey responses.

As per Leger’s data storage and transfer protocols, your personal contact information will be initially collected on Leger’s data server in Montreal before being transferred to our study team.

**D22. Are you interested in being contacted about follow up research conducted by our research team on topics related to COVID-19 vaccination that were presented in our survey?**

**O** Yes, I consent to provide my contact information to learn about future research studies

**O** No, I do not wish to be contacted to learn about future studies.

[IF D22=“I CONSENT” ASK D23 and D24, ELSE SKIP TO CLOSE]

D23. Thank you for your interest. Please provide an email address where a member of our research team can contact you:

**[**RECORD EMAIL ADDRESS. ENSURE VALID EMAIL FORMAT.]

D24. Could we please have your first name?

[RECORD FIRST NAME]

Thank you for your participation.

**SUBMIT**

***Your participation provides important insights that will be used to help improve the COVID-19 pandemic response. For more information about COVID-19 please visit please visit:*** [***www.Canada.ca/covid-19***](http://www.Canada.ca/covid-19)***.***
