# Supplementary material for: Factors affecting hesitancy toward COVID-19 vaccine booster doses in Canada: a cross-national survey
Source: Can J Public Health. 2023 Nov 22;115(1):26–39. doi: 10.17269/s41997-023-00823-z (PMC10853155; doi:10.17269/s41997-023-00823-z)
Supplement: Supplementary file 3 — Supplementary file3 (DOCX 539 KB) [file 41997_2023_823_MOESM3_ESM.docx]

**Supplemental File 3**

**Table of Contents**

**Figure 1 Participant responding “agree” with statements regarding their self-reported ability to examine health information**

**Figure 2 Top reasons for brand preference in respondents COVID-19 booster dose.**

**Figure 3 Top reasons for brand preference in respondents first COVID-19 vaccine dose.**

**Supplemental File 3, Figure 4 Top reasons for brand preference in respondents second COVID-19 vaccine dose.**

**Figure 5 The perceived effectiveness of COVID-19 vaccines for preventing infection from all variants of concern (VOCs), between first and second dose recipients.**

**Figure 6 The perceived effectiveness of COVID-19 vaccines for preventing serious illness from all variants of concern (VOCs), between first and second dose recipients.**

**Table 1 Exemplary quotations of top reasons for COVID-19 booster dose brand preference.**

**Table 2 Odds of experiencing first dose vaccine hesitancy given sociodemographics [All independent variables fitted]**

**Table 3 Odds of experiencing first dose vaccine hesitancy given trust and beliefs [All independent variables fitted]**

**Table 4 Odds of experiencing booster dose vaccine hesitancy given sociodemographics [All independent variables fitted]**

**Table 5 Odds of experiencing booster dose vaccine hesitancy given trust and beliefs [All independent variables fitted]**

**Table 6 Odds of experiencing first dose vaccine hesitancy given sociodemographics [Sidak correction]**

**Table 7 Odds of experiencing booster dose vaccine hesitancy given sociodemographics [Sidak correction]**

**Table 8 Odds of experiencing first dose vaccine hesitancy given trust and beliefs [Sidak correction]**

**Table 9 Odds of experiencing booster dose vaccine hesitancy given trust and beliefs [Sidak correction]**

**
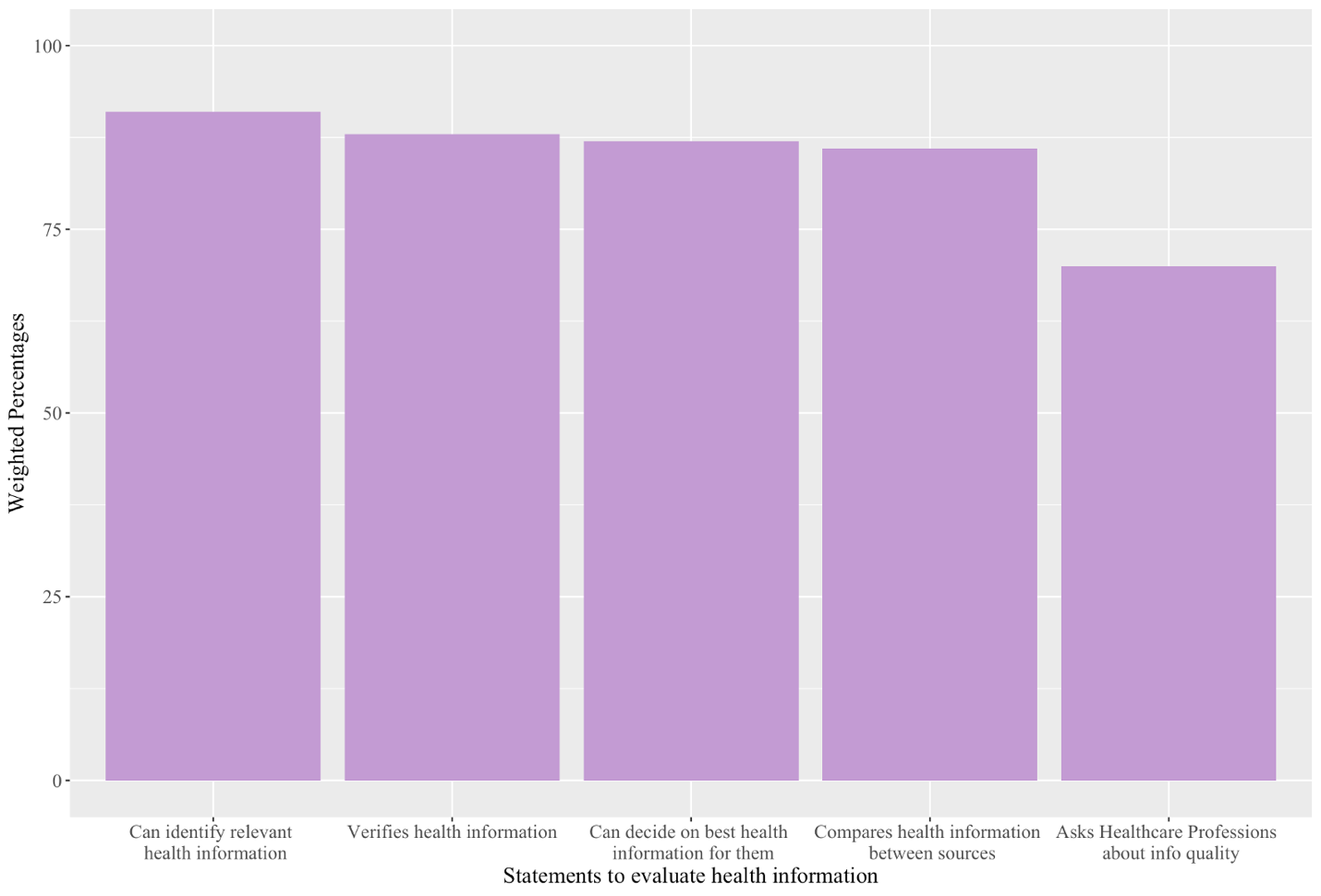
**

Response options included ‘completely disagree’, ‘somewhat disagree’, ‘somewhat agree’, and ‘completely agree’ which were to the questions ‘I can identify if health information is relevant to me or not’, ‘When I hear about or read about new health information I verify if it is true or not’, ‘I decide what health information is best for me’, ‘I compare health information from different sources’, and ‘I ask a health professional about the quality of information I find’. Response options included ‘completely disagree’, ‘somewhat disagree’, ‘somewhat agree’, and ‘completely agree’. ‘Completely agree’ and ‘somewhat agree’ were combined into ‘agree’.

**Supplemental File 3, Figure 1** Participant responding “agree” with statements regarding their self-reported ability to examine health information.


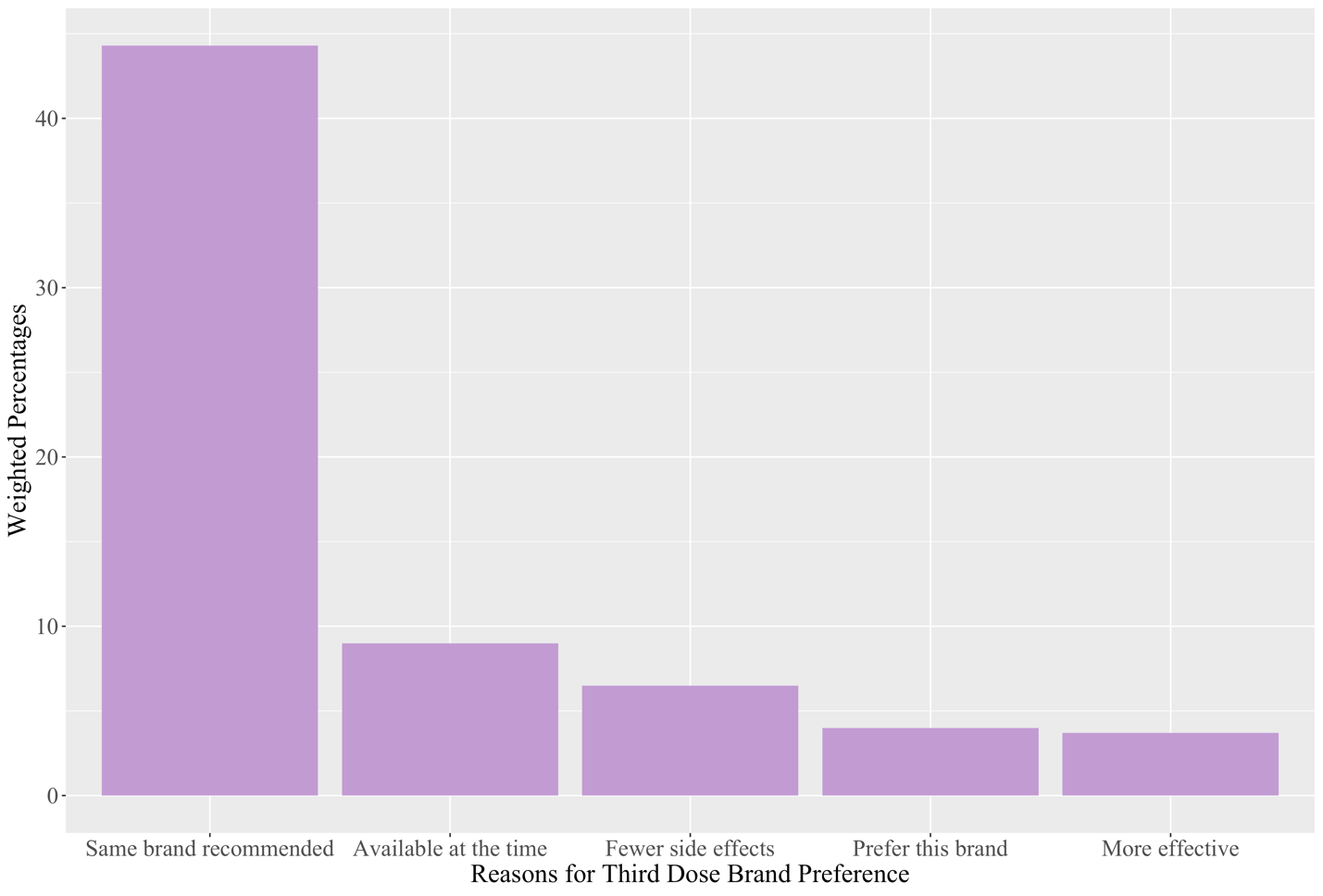


Brand preference categories were coded to ‘Briefly describe (1-3 sentences) in your own words the reason(s) behind your vaccine brand preference for your third dose’. Responses were open-ended and coded into qualitative categories by Leger. Top five most frequent answers are reported. Prefer not to answer responses were excluded.

**Supplemental File 3 Figure 2** Top reasons for brand preference in respondents COVID-19 booster dose.

**
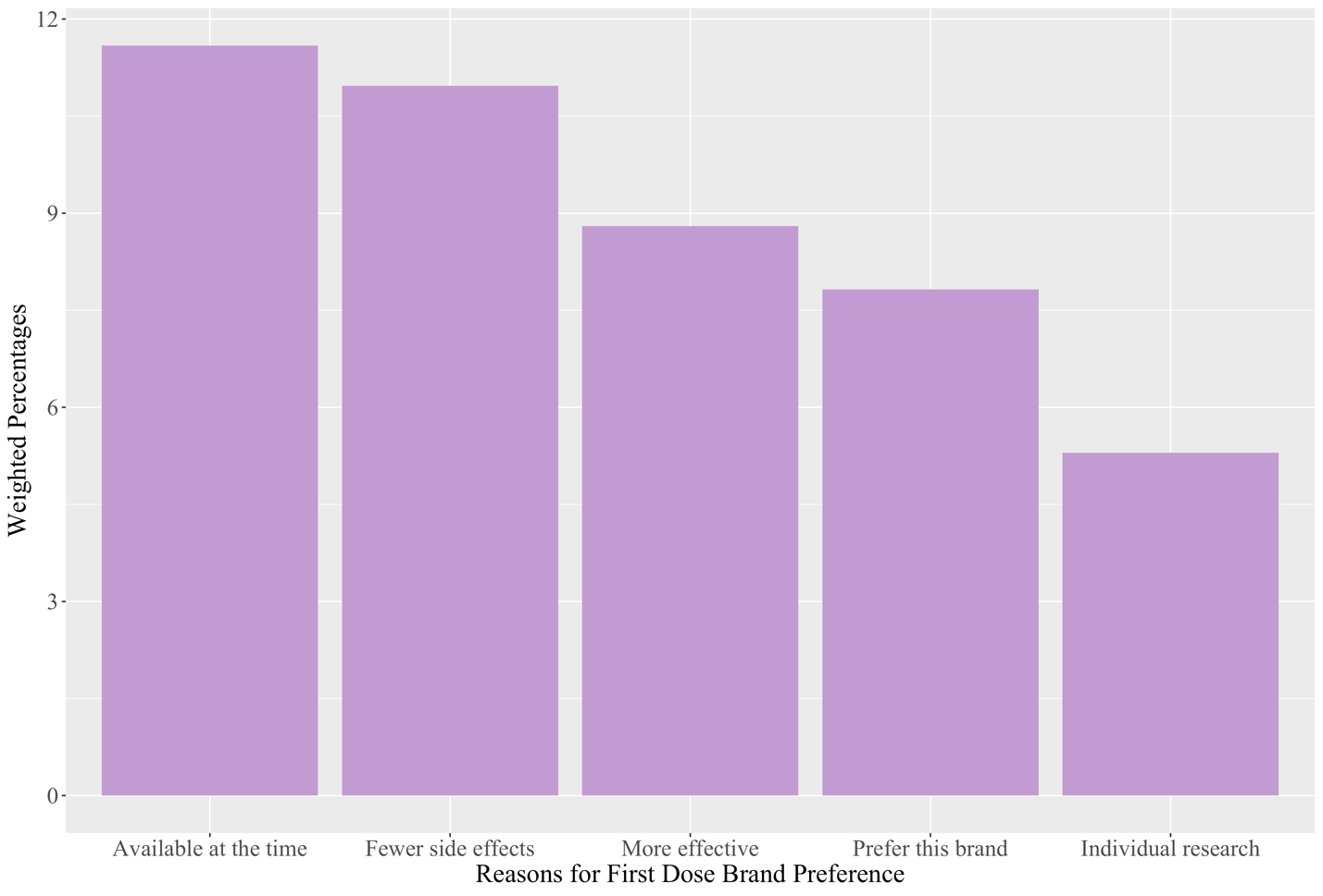
**

Brand preference categories were coded to ‘Briefly describe (1-3 sentences) in your own words the reason(s) behind your vaccine brand preference for your first dose’. Responses were open-ended and coded into qualitative categories by Leger. Top five most frequent answers are reported. Prefer not to answer responses were excluded.

**Supplemental File 3, Figure 3** Top reasons for brand preference in respondents first COVID-19 vaccine dose.

**
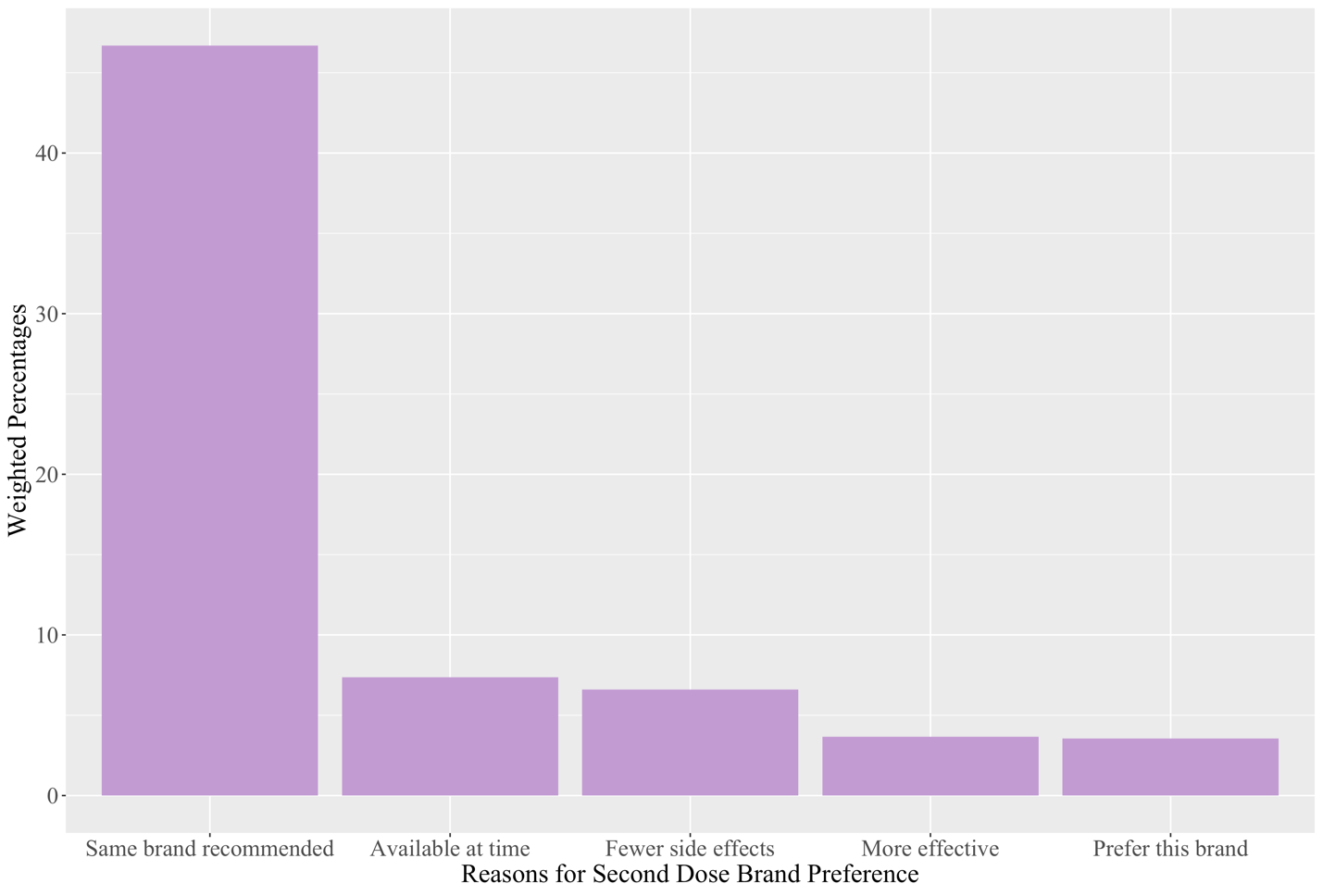
**

Brand preference categories were coded to ‘Briefly describe (1-3 sentences) in your own words the reason(s) behind your vaccine brand preference for your second dose’. Responses were open-ended and coded into qualitative categories by Leger. Top five most frequent answers are reported. Prefer not to answer responses were excluded.

**Supplemental File 3, Figure 4** Top reasons for brand preference in respondents second COVID-19 vaccine dose.


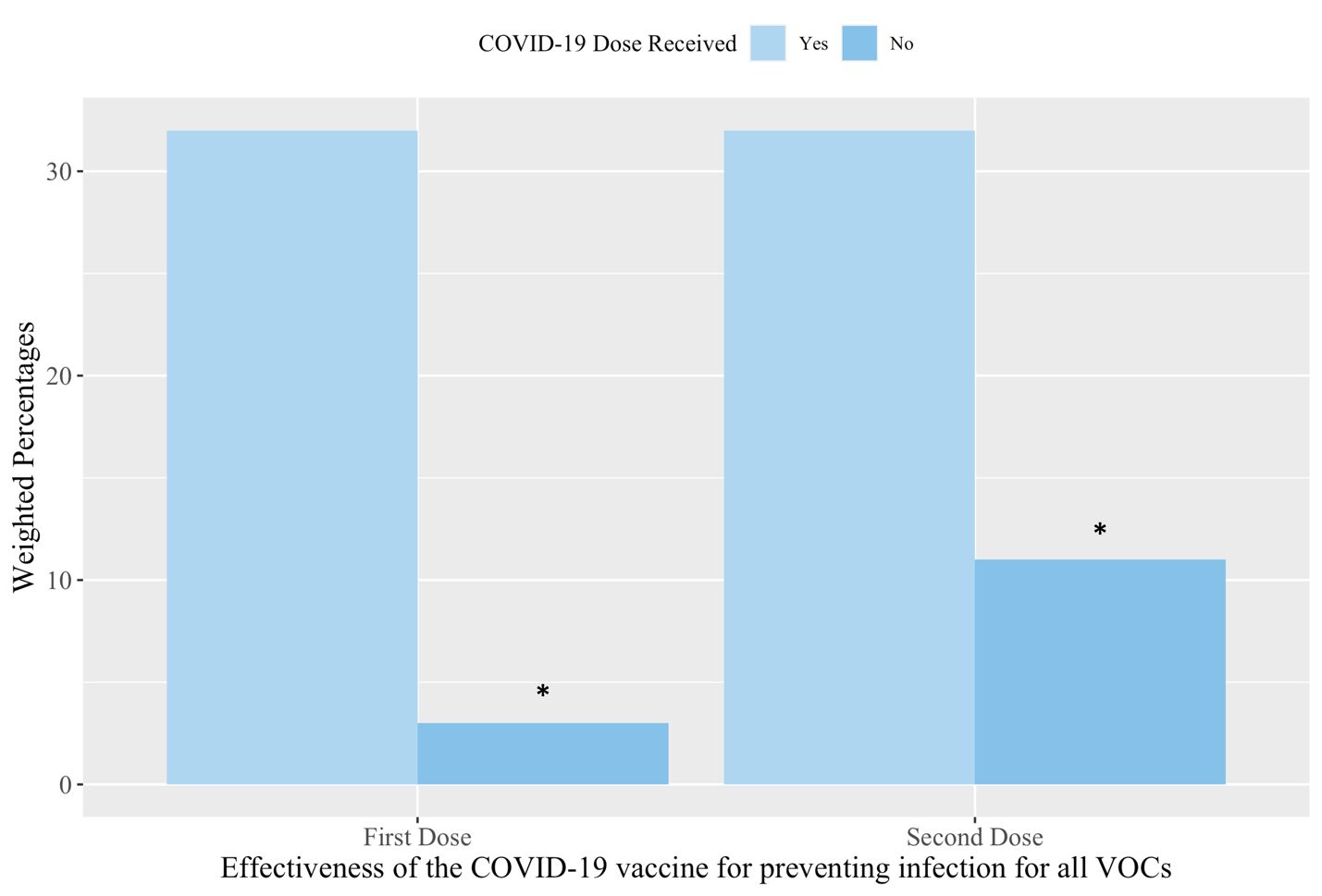


Response options applied to the survey question *‘In your opinion, are vaccines effective at preventing infection from the SARS-CoV-2 virus (the virus that causes COVID-19 disease)?*

* Indicates p≤0.01 when compared to the ‘Yes’ group of the same dose

Abbreviations: Variants of concern (VOCs)

**Supplemental File 3, Figure 5** The perceived effectiveness of COVID-19 vaccines for preventing infection from all variants of concern (VOCs), between first and second dose recipients.


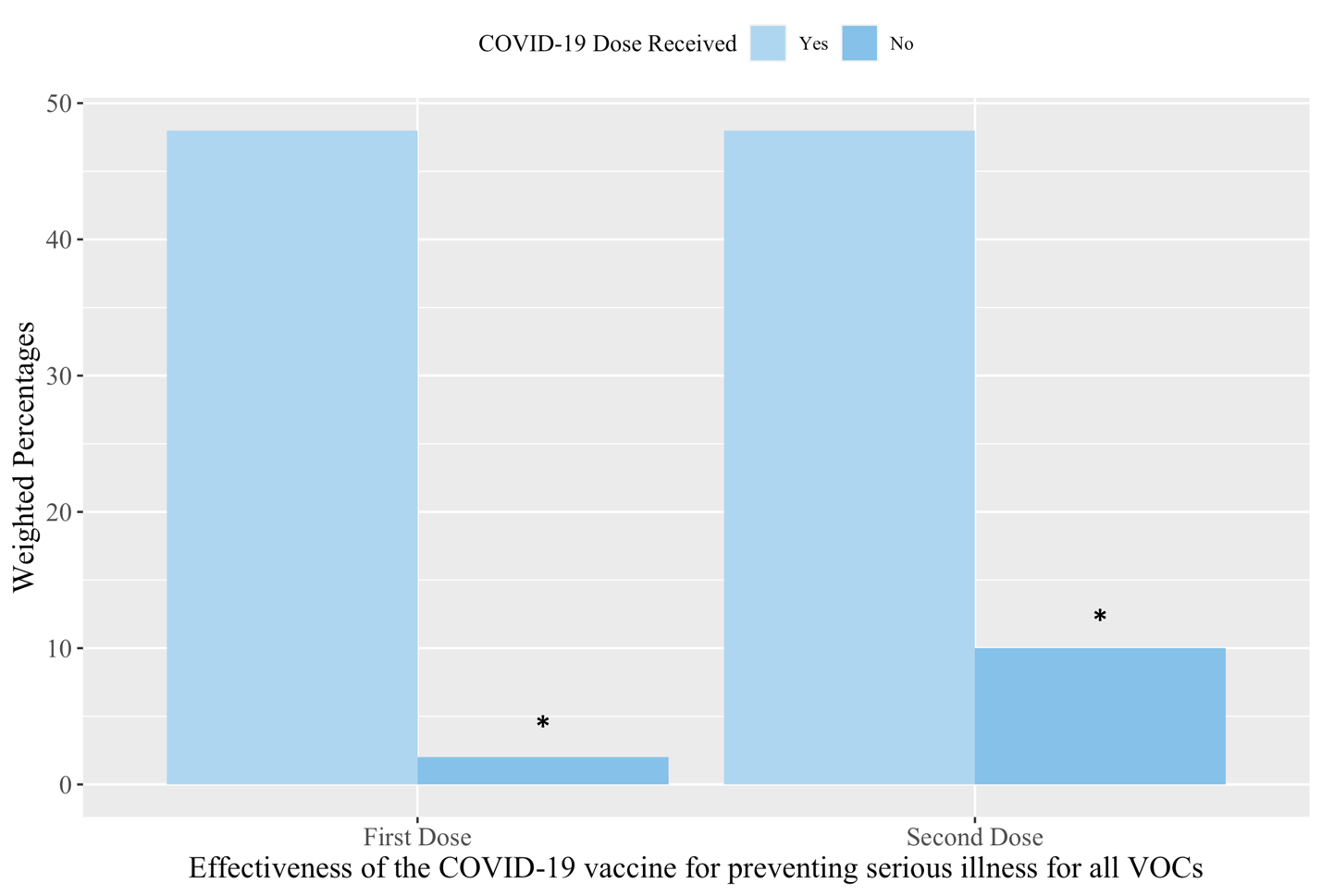


Response options applied to the survey question *‘In your opinion, are vaccines effective at preventing serious illness from the SARS-CoV-2 virus (the virus that causes COVID-19 disease)?*

* Indicates p≤0.01 when compared to the ‘Yes’ group of the same dose

Abbreviations: Variants of concern (VOCs)

**Supplementary File 3, Figure 6** The perceived effectiveness of COVID-19 vaccines for preventing serious illness from all variants of concern (VOCs), between first and second dose recipients.

**Supplemental File 3, Table 1 Exemplary quotations of top reasons for COVID-19 booster dose brand preference**.

| **Qualitative Categories** | **Exemplary Quotations** |
| --- | --- |
| Recommendations to stay with the same brand/preferred not to mix/consistency | “It [staying with the same vaccine brand] was recommended after 2 Pfizer”  “I wish to keep all vaccines within the same brand to maximize potential results, and minimize potential downfalls”  “Je voulais trois doses pareilles” |
| Offered or available at the time | “Only one offered”  “Did not have a choice, what was available”  “Would have taken either but my clinic offered only Moderna at the time I went” |
| Fewer side effects | “Less side effects for those under 30”  “Effets secondairas minimes”  “Because of the least side effects” |
| Preferred the brand | “My preference was Pfizer and that was the one that I chose and received”  “Si je le fais, ce sera Moderna”  "It was the best one I found after doing research” |
| More effective | “C’était la forte qu’il avait vu de”  “I read online that Pfizer is more effective”  “Discussions about higher efficacy of the Moderna vaccine for the third dose” |

Exemplary quotations to the open-ended question: “Briefly describe (1-3 sentences) in your own words the reason(s) behind your vaccine brand preference for your third dose.”

**Supplemental File 3, Table 2 Odds of experiencing first dose vaccine hesitancy given sociodemographics**

| **Covariate** | **Categories** | **Overall p-value** | **Strata**  **p-value** | **OR**  **(Adjusted)** | **95% CI** |
| --- | --- | --- | --- | --- | --- |
| Age |  | 0.048 | 0.048 | 0.987 | 0.975, 1.000 |
| Income | $0-$49,999 (Reference Category) | 0.015 |  | | |
|  | $50,000-$99,999 |  | 0.152 | 0.678 | 0.398, 1.155 |
|  | $100,000-$149,999 |  | 0.003 | 0.342 | 0.170, 0.688 |
|  | $150,000-or more |  | 0.034 | 0.401 | 0.172, 0.933 |
| Education | College/University Degree (Reference Category) | 0.579 |  | | |
|  | High school or less |  | 0.204 | 1.492 | 0.805, 2.767 |
|  | CEGEP/Vocational college/Trade |  | 0.486 | 1.274 | 0.645, 2.517 |
|  | Some College or University (no degree) |  | 0.939 | 1.028 | 0.505, 2.094 |
| With child(ren) under the age of 18 years | No (Reference Category) | <0.001 |  | | |
|  | Yes |  | <0.001 | 2.398 | 1.473, 3.905 |
| Region | Ontario (Reference Category) | 0.022 |  | | |
|  | British Columbia |  | 0.028 | 1.906 | 1.071, 3.394 |
|  | Alberta |  | 0.698 | 1.155 | 0.556, 2.401 |
|  | Saskatchewan/Manitoba |  | 0.100 | 0.398 | 0.133, 1.195 |
|  | Quebec |  | 0.239 | 0.674 | 0.349, 1.300 |
|  | Atlantic |  | 0.732 | 0.819 | 0.261, 2.573 |
|  | Territories |  | 0.207 | 3.199 | 0.525, 19.51 |
| Lived in Canada | <5 years (Reference Category) | 0.844 |  |  |  |
|  | 5-<10 years |  | 0.651 | 1.329 | 0.388, 4.558 |
|  | 10-<10 years |  | 0.861 | 1.128 | 0.293, 4.333 |
|  | >20 years |  | 0.516 | 1.483 | 0.451, 4.871 |
| Sex | Female (Reference Category) | 0.048 |  |  |  |
|  | Male |  | 0.048 | 1.540 | 1.004, 2.362 |
| Ethnicity | White (Reference Category) | 0.309 |  |  |  |
|  | Asian East/Southeast |  | 0.066 | 0.473 | 0.213, 1.052 |
|  | Asian South/Indian Caribbean |  | 0.104 | 0.274 | 0.057, 1.305 |
|  | Black |  | 0.279 | 1.631 | 0.672, 3.958 |
|  | Indigenous |  | 0.825 | 1.156 | 0.318, 4.205 |
|  | Latin American |  | 0.662 | 0.623 | 0.075, 5.194 |
|  | Middle Eastern |  | 0.684 | 0.652 | 0.083, 5.132 |
|  | Mixed/Other |  | 0.619 | 0.752 | 0.245, 2.308 |

Logistic regression model selection with all independent variables fitted. Response option “Prefer not to answer” from each independent variable were excluded from the dataset.

**Supplemental File 3, Table 3 Odds of experiencing first dose vaccine hesitancy given trust and beliefs**

| **Covariate** | **Categories** | **Overall p-value** | **Strata**  **p-value** | **OR**  **(Adjusted)** | **95% CI** |
| --- | --- | --- | --- | --- | --- |
| Effectiveness of vaccines in preventing infection | Yes, to all VOCs (RC) | 0.112 |  | | |
|  | Yes, to some VOCs |  | 0.676 | 1.328 | 0.350, 5.044 |
|  | No, to no VOCs |  | 0.146 | 2.678 | 0.710, 10.10 |
|  | Other |  | 0.691 | 0.568 | 0.035, 9.287 |
| Effectiveness of vaccines at preventing serious illness | Yes, to all VOCs (RC) | <0.001 |  | | |
|  | Yes, to some VOCs |  | 0.001 | 12.99 | 2.942, 57.37 |
|  | No, to no VOCs |  | <0.001 | 45.94 | 10.25, 205.9 |
|  | Other |  | 0.001 | 31.58 | 3.955, 252.1 |
| Possibility to be vaccinated too many times | No (RC) | 0.007 |  | | |
|  | Yes |  | 0.007 | 2.255 | 1.242, 4.095 |
| Trust the pharmaceutical industries | Strongly agree (RC) | 0.304 |  | | |
|  | Somewhat agree |  | 0.210 | 0.356 | 0.071, 1.791 |
|  | Unsure/no opinion |  | 0.344 | 0.481 | 0.105, 2.193 |
|  | Somewhat disagree |  | 0.342 | 0.474 | 0.102, 2.208 |
|  | Strongly disagree |  | 0.772 | 0.793 | 0.165, 3.808 |
| Trust the federal government is making decisions in best interest | Strongly agree (RC) | 0.497 |  | | |
|  | Somewhat agree |  | 0.275 | 5.029 | 0.277, 91.33 |
|  | Unsure/no opinion |  | 0.156 | 8.312 | 0.446, 154.8 |
|  | Somewhat disagree |  | 0.258 | 5.261 | 0.296, 93.58 |
|  | Strongly disagree |  | 0.184 | 7.327 | 0.388, 138.4 |
| Trust the provincial government is making decisions in best interest | Strongly agree (RC) | 0.136 |  | | |
|  | Somewhat agree |  | 0.592 | 0.548 | 0.061, 4.933 |
|  | Unsure/no opinion |  | 0.506 | 0.492 | 0.061, 3.992 |
|  | Somewhat disagree |  | 0.816 | 1.275 | 0.164, 9.942 |
|  | Strongly disagree |  | 0.493 | 2.066 | 0.259, 16.51 |

Logistic regression model selection with all independent variables fitted. Response option “Prefer not to answer” from each independent variable were excluded from the dataset.

**Supplemental File 3, Table 4 Odds of experiencing booster dose vaccine hesitancy given sociodemographics**

| **Covariate** | **Categories** | **Overall p-value** | **Strata**  **p-value** | **OR**  **(Adjusted)** | **95% CI** |
| --- | --- | --- | --- | --- | --- |
| Age | (Continuous) | <0.001 |  | 0.952 | 0.942, 0.961 |
| Income | $0-$49,999 (Reference Category) | 0.024 |  | | |
|  | $50,000-$99,999 |  | 0.241 | 0.805 | 0.561, 1.157 |
|  | $100,000-$149,999 |  | 0.022 | 0.604 | 0.393, 0.930 |
|  | $150,000-or more |  | 0.007 | 0.479 | 0.280, 0.819 |
| Education | College/University Degree (Reference Category) | 0.001 |  | | |
|  | High school or less |  | 0.001 | 1.933 | 1.301, 2.872 |
|  | CEGEP/Vocational college/Trade |  | 0.010 | 1.844 | 1.160, 2.933 |
|  | Some College or University (no degree) |  | 0.699 | 0.913 | 0.576, 1.447 |
| With child(ren) under the age of 18 years | No (Reference Category) | <0.001 |  | | |
|  | Yes |  | <0.001 | 1.876 | 1.368, 2.572 |
| Region | Ontario (Reference Category) | <0.001 |  | | |
|  | British Columbia |  | 0.774 | 1.068 | 0.682, 1.671 |
|  | Alberta |  | 0.012 | 1.804 | 1.140, 2.854 |
|  | Saskatchewan/Manitoba |  | 0.772 | 1.098 | 0.538, 2.068 |
|  | Quebec |  | 0.545 | 0.874 | 0.565,1.351 |
|  | Atlantic |  | 0.217 | 1.430 | 0.810, 2.524 |
|  | Territories |  | <0.001 | <0.001 | 0.000, 0.000 |
| Lived in Canada | <5 years (Reference Category) | 0.673 |  |  |  |
|  | 5-<10 years |  | 0.924 | 0.967 | 0.492, 1.901 |
|  | 10-<10 years |  | 0.491 | 1.323 | 0.597, 2.931 |
|  | >20 years |  | 0.414 | 1.291 | 0.699, 2.385 |
| Sex | Female (Reference Category) | 0.223 |  |  |  |
|  | Male |  | 0.223 | 1.197 | 0.897, 1.598 |
| Ethnicity | White (Reference Category) | 0.476 |  |  |  |
|  | Asian East/Southeast |  | 0.639 | 0.894 | 0.560, 1.427 |
|  | Asian South/Indian Caribbean |  | 0.532 | 1.231 | 0.640, 2.365 |
|  | Black |  | 0.087 | 1.766 | 0.920, 3.392 |
|  | Indigenous |  | 0.981 | 1.012 | 0.385, 2.663 |
|  | Latin American |  | 0.702 | 1.250 | 0.398, 3.930 |
|  | Middle Eastern |  | 0.099 | 2.504 | 0.840, 7.465 |
|  | Mixed/Other |  | 0.336 | 1.392 | 0.709, 2.735 |

Logistic regression model selection with all independent variables fitted. Response option “Prefer not to answer” from each independent variable were excluded from the dataset.

**Supplemental File 3, Table 5 Odds of experiencing booster dose vaccine hesitancy given trust and beliefs**

| **Covariate** | **Categories** | **Overall p-value** | **Strata**  **p-value** | **OR**  **(Adjusted)** | | **95% CI** | |
| --- | --- | --- | --- | --- | --- | --- | --- |
| Effectiveness of vaccines at preventing infection | Yes, to all VOCs (Reference Category) | <0.001 |  | | | | |
|  | Yes, to some VOCs |  | 0.407 | 1.239 | | 0.747, 2.054 | |
|  | No, to no VOCs |  | <0.001 | 3.693 | | 1.976, 6.900 | |
|  | Other |  | 0.019 | 4.011 | | 1.251, 12.856 | |
| Effectiveness of vaccines at preventing serious illness | Yes, to all VOCs (Reference Category) | 0.002 |  | | | | |
|  | Yes, to some VOCs |  | 0.007 | 1.773 | | 1.173, 2.679 | |
|  | No, to no VOCs |  | <0.001 | 3.149 | | 1.693, 5.857 | |
|  | Other |  | 0.232 | 2.076 | | 0.626, 6.884 | |
| Possibility to be vaccinated too many times | No (Reference Category) | <0.001 |  | | | | |
|  | Yes |  | <0.001 | 2.070 | | 1.533, 2.796 | |
| Trust the motives of pharmaceutical industries | Strongly agree (Reference Category) | 0.263 |  | | | | |
|  | Somewhat agree |  | 0.242 | | 0.689 | | 0.369, 1.286 |
|  | Unsure/no opinion |  | 0.047 | | 0.533 | | 0.286, 0.993 |
|  | Somewhat disagree |  | 0.329 | | 0.720 | | 0.372, 1.393 |
|  | Strongly disagree |  | 0.348 | | 0.704 | | 0.338, 1.466 |
| Trust the Canadian federal government is making decisions in best interest | Strongly agree (Reference Category) | <0.001 |  | | | | |
|  | Somewhat agree |  | 0.086 | 1.645 | | 0.932, 2.936 | |
|  | Unsure/no opinion |  | 0.112 | 1.728 | | 0.880, 3.391 | |
|  | Somewhat disagree |  | 0.002 | 2.924 | | 1.504, 5.684 | |
|  | Strongly disagree |  | <0.001 | 5.100 | | 2.435, 10.68 | |
| Trust the provincial government is making decisions in best interest | Strongly agree (Reference Category) | 0.002 |  | | | | |
|  | Somewhat agree |  | 0.026 | 2.025 | | 1.088, 3.771 | |
|  | Unsure/no opinion |  | 0.001 | 3.462 | | 1.698, 7.061 | |
|  | Somewhat disagree |  | 0.219 | 1.533 | | 0.776, 3.031 | |
|  | Strongly disagree |  | 0.569 | 1.257 | | 0.573, 2.759 | |

Logistic regression model selection with all independent variables fitted. Response option “Prefer not to answer” from each independent variable were excluded from the dataset.

**Table 6 Odds of experiencing first dose vaccine hesitancy given sociodemographics [Sidak correction]**

| **Covariate** | **Categories** | **Overall p-value** | **Strata**  **p-value** | **OR**  **(Adjusted)** | **95% CI** |
| --- | --- | --- | --- | --- | --- |
| Income | $0-$49,999 (Reference Category) | **0.002** |  | | |
|  | $50,000-$99,999 |  | 0.043 | 0.605 | 0.372, 0.984 |
|  | $100,000-$149,999 |  | **0.001** | 0.334 | 0.175, 0.639 |
|  | $150,000-or more |  | **0.010** | 0.366 | 0.170, 0.787 |
| With child(ren) under the age of 18 years | No (Reference Category) | **<0.001** |  | | |
|  | Yes |  | **<0.001** | 2.318 | 1.504, 3.574 |

Logistic regression model selection was conducted from the fitted model using backward stepwise selection with elimination stopping rule to a p-value <0.01. The Sidak correction was applied and bolded numbers are considered statistically significant results with a corrected alpha of 0.02.

**Table 7 Odds of experiencing booster dose vaccine hesitancy given sociodemographics [Sidak correction]**

| **Covariate** | **Categories** | **Overall p-value** | **Strata**  **p-value** | **OR**  **(Adjusted)** | **95% CI** |
| --- | --- | --- | --- | --- | --- |
| Age | (Continuous) | **<0.001** |  | 0.951 | 0.942, 0.961 |
| Education | College/University Degree (Reference Category) | **0.002** |  | | |
|  | High school or less |  | **0.001** | 1.903 | 1.291, 2.806 |
|  | CEGEP/Vocational college/Trade |  | **0.009** | 1.859 | 1.168, 2.959 |
|  | Some College or University (no degree) |  | 0.781 | 0.938 | 0.598, 1.472 |
| With child(ren) under the age of 18 years | No (Reference Category) | **<0.001** |  | | |
|  | Yes |  | **<0.001** | 1.889 | 1.389, 2.570 |

Logistic regression model selection was conducted from the fitted model using backward stepwise selection with elimination stopping rule to a p-value <0.01. The Sidak correction was applied and bolded numbers are considered statistically significant results with a corrected alpha of 0.01.

**Table 8 Odds of experiencing first dose vaccine hesitancy given trust and beliefs [Sidak correction]**

| **Covariate** | **Categories** | **Overall p-value** | **Strata**  **p-value** | **OR**  **(Adjusted)** | **95% CI** |
| --- | --- | --- | --- | --- | --- |
| Effectiveness of vaccines at preventing serious illness | Yes, to all VOCs (RC) | **<0.001** |  | | |
|  | Yes, to some VOCs |  | **<0.001** | 16.19 | 3.683, 71.20 |
|  | No, to no VOCs |  | **<0.001** | 54.08 | 12.09, 241.9 |
|  | Other |  | **<0.001** | 49.91 | 6.043, 364.2 |
| Possibility to be vaccinated too many times | No (RC) | **0.003** |  | | |
|  | Yes |  | **0.003** | 2.496 | 1.365, 4.562 |
| Trust the provincial government is making decisions in best interest | Strongly agree (RC) | **<0.001** |  | | |
|  | Somewhat agree |  | 0.834 | 1.165 | 0.280, 4.857 |
|  | Unsure/no opinion |  | 0.527 | 1.592 | 0.376, 6.737 |
|  | Somewhat disagree |  | 0.105 | 3.252 | 0.781, 13.54 |
|  | Strongly disagree |  | **0.003** | 7.901 | 2.003, 31.16 |

Logistic regression model selection was conducted from the fitted model using backward stepwise selection with elimination stopping rule to a p-value <0.01. The Sidak correction was applied and bolded numbers are considered statistically significant results with a corrected alpha of 0.01.

**Table 9 Odds of experiencing booster dose vaccine hesitancy given trust and beliefs [Sidak correction]**

| **Covariate** | **Categories** | **Overall p-value** | **Strata**  **p-value** | **OR**  **(Adjusted)** | **95% CI** |
| --- | --- | --- | --- | --- | --- |
| Effectiveness of vaccines at preventing infection | Yes, to all VOCs (Reference Category) | **<0.001** |  | | |
|  | Yes, to some VOCs |  | 0.407 | 1.239 | 0.747, 2.054 |
|  | No, to no VOCs |  | **<0.001** | 3.693 | 1.976, 6.900 |
|  | Other |  | 0.019 | 4.011 | 1.251, 12.86 |
| Effectiveness of vaccines at preventing serious illness | Yes, to all VOCs (Reference Category) | **0.002** |  | | |
|  | Yes, to some VOCs |  | **0.007** | 1.773 | 1.173, 2.679 |
|  | No, to no VOCs |  | **<0.001** | 3.149 | 1.693, 5.857 |
|  | Other |  | 0.232 | 2.076 | 0.626, 6.884 |
| Possibility to be vaccinated too many times | No (Reference Category) | **<0.001** |  | | |
|  | Yes |  | **<0.001** | 2.070 | 1.533, 2.796 |
| Trust the Canadian federal government is making decisions in best interest | Strongly agree (Reference Category) | **<0.001** |  | | |
|  | Somewhat agree |  | 0.179 | 1.496 | 0.830, 2.697 |
|  | Unsure/no opinion |  | 0.237 | 1.522 | 0.759, 3.052 |
|  | Somewhat disagree |  | **0.004** | 2.697 | 1.376, 5.285 |
|  | Strongly disagree |  | **<0.001** | 4.624 | 2.202, 9.710 |
| Trust the provincial government is making decisions in best interest | Strongly agree (Reference Category) | **0.008** |  | | |
|  | Somewhat agree |  | 0.035 | 1.930 | 1.046, 3.562 |
|  | Unsure/no opinion |  | **0.001** | 3.131 | 1.547, 6.336 |
|  | Somewhat disagree |  | 0.234 | 1.497 | 0.770, 2.910 |
|  | Strongly disagree |  | 0.590 | 1.234 | 0.574, 2.649 |

Logistic regression model selection was conducted from the fitted model using backward stepwise selection with elimination stopping rule to a p-value <0.01. The Sidak correction was applied and bolded numbers are considered statistically significant results with a corrected alpha of 0.01.
